# Guidelines on reporting and assessing dynamic mathematical models of infectious diseases: a scoping review

**DOI:** 10.1186/s12879-025-12211-8

**Published:** 2025-12-31

**Authors:** Madhav Chaturvedi, Antonia Bartz, Claudia M. Denkinger, Carolina Klett-Tammen, Mirjam Kretzschmar, Alexander Kuhlmann, Berit Lange, Florian M. Marx, Rafael Mikolajczyk, Ina Monsef, Hoa Thi Nguyen, Janik Suer, Nicole Skoetz, Veronika K. Jaeger, André Karch

**Affiliations:** 1 Institute of Epidemiology and Social Medicine, University of Münster, Münster, Germany; 2 Division of Infectious Disease and Tropical Medicine, Partner Site Heidelberg, University Hospital Heidelberg & German Center for Infection Research, Heidelberg, Germany; 3 Department of Epidemiology, Helmholtz Centre for Infection Research (HZI), Braunschweig, Germany; 4 Department of Epidemiology, University Medical Center Utrecht, Utrecht University, Utrecht, The Netherlands; 5 Center for Complex Systems Studies (CCSS), Utrecht University, Utrecht, The Netherlands; 6 University Clinic Schleswig-Holstein, University of Lübeck, Lübeck, Germany; 7 Institute for Infectious Disease Epidemiology, TWINCORE, Hannover, Lower Saxony, Germany; 8 South African Centre for Epidemiological Modelling and Analysis (SACEMA), Stellenbosch University, Stellenbosch, South Africa; 9 Institute for Medical Epidemiology, Biometrics, and Informatics, Interdisciplinary Center for Health Sciences, Medical Faculty of the Martin, Luther University Halle-Wittenberg, Halle, Germany; 10 Institute of Public Health, Medical Faculty and University Hospital Cologne, University of Cologne, Cologne, Germany; 11 Medical Faculty and University Hospital Heidelberg, Heidelberg Institute of Global Health, University of Heidelberg, Heidelberg, Germany

**Keywords:** Infectious disease modelling, Quality assessment, Reporting guideline

## Abstract

**Background:**

Mathematical models are valuable tools for guiding public health policy decisions to combat the spread of infectious diseases. Nevertheless, a lack of appropriate quality assessment tools and reporting guidelines hinders the comprehensibility, transparency, and credibility of infectious disease modelling studies and the ability to assess their quality. In a first step towards addressing the need for reporting guidelines and quality assessment tools specific to infectious disease modelling, this scoping review identified common themes in existing reporting and quality assessment guidance for infectious disease modelling studies and adjacent fields.

**Methods:**

We conducted temporally-unrestricted searches on Medline (via Ovid), Web of Science, medRxiv, and bioRxiv in January 2024 to find articles that provide guidance on writing or assessing modelling studies within infectious disease modelling and adjacent fields including but not limited to healthcare and, more specifically, health economics. Articles were double-screened for eligibility via title-and-abstract screening and full-text screening. Recommendations made by eligible articles were classified into 31 subdimensions which were categorised into seven overarching dimensions (*1. applicability*; 2. *model structure*; 3. *parameterisation and calibration*; 4. *validity*; 5. *uncertainty*; 6. *interpretation*; 7. *reproducibility*,* clarity*,* and transparency*). We followed the PRISMA extension for reporting scoping reviews.

**Results:**

Our final review included 53 articles. All dimensions except for *interpretation* were covered by most articles (81%-98%). However, we found substantial heterogeneity in the frequency with which subdimensions were addressed (11%-96%). Subdimensions pertaining to parameter uncertainty and transparency about parameter values were mentioned in most articles (91%-96%); conversely, discussions about auxiliary publication details and software implementation were covered less frequently (11%-23%).

**Conclusions:**

This review shows that many recommendations made by reporting guidelines and quality assessment tools have thematic similarities and address common topics that are also relevant to infectious disease modelling. These identified themes and recommendations can be used as a starting point to inform the development of standardised guidelines for infectious disease modelling.

**Registration DOI:**

10.17605/OSF.IO/AB6D3.

**Clinical trial number:**

Not applicable.

**Supplementary Information:**

The online version contains supplementary material available at 10.1186/s12879-025-12211-8.

## Background

Infectious diseases remain a key cause of morbidity and mortality worldwide [1]. Nowadays, mathematical modelling is increasingly used to study the dynamics, transmission, and control of infectious diseases. The role of modelling as an indispensable tool for guiding public health policy was exemplified during the COVID-19 pandemic [2]. Modelling fields beyond infectious disease modelling have undertaken considerable efforts to standardise reporting and quality assessment of modelling studies [3, 4]. However, when attempting to gather evidence to develop recommendations for policy-relevant decisions during the COVID-19 pandemic, our research team had difficulties identifying any quality assessment tools with which we could assess the quality and risk of bias of modelling studies. Furthermore, in a random subset of 100 recently published infectious disease modelling studies, only one of the 100 studies reported using a guideline for their publication (details on this search can be found in subsection 1.2 of Additional File 1). This guideline was not specific to infectious disease modelling, but was instead a guideline on reporting agent-based models agnostic to field of application [5]. This leads us to believe that there is a lack of widely-used guidelines for the reporting and assessment of infectious disease modelling studies. This is a recognised issue which is also being addressed by the Lancet Commission on Strengthening the Use of Epidemiological Modelling of Emerging and Pandemic Infectious Diseases [6].

Inadequate reporting of research and a lack of standard quality assessment methods can lead to misinterpretation and hinder the ability to assess the validity of a study. Reporting guidelines and quality assessment tools are instruments that aim to counteract these shortcomings [7]. Reporting guidelines provide structured frameworks to transparently report study methods, results, and interpretations, ensuring that key information is consistently reported [8, 9]. Quality assessment tools like the Cochrane Tool for assessing RCTs [10] enable a systematic evaluation of studies by identifying potential sources of bias or systematic error. Adhering to reporting guidelines allows researchers to enhance the transparency, reliability, and credibility of their research. Conducting quality assessments enables readers, researchers, and policy makers to critically assess the reliability and validity of study results and directly compare different studies addressing the same key question.

Having standardised reporting guidelines and quality assessment tools specifically for infectious disease modelling studies would help researchers, public health authorities, and decisionmakers to better judge and assess evidence from modelling studies, ultimately contributing to more comprehensive understanding of infection dynamics, transmission, and control strategies.

For these reasons, we embarked on the project of creating such standardised reporting guidelines and/or quality assessment tools. As a first step, we decided to investigate what literature already exists in this area and what recommendations have been made. In this scoping review, we identified existing guidance and best practice recommendations—for reporting and/or assessing studies—in the field of infectious disease modelling, as well as in adjacent fields. Specifically, we determined common themes across the identified publications which could be used in a next step following this review to create a core item set for a standardised reporting guideline and quality assessment tool—or a suite of such tools with a common origin—specific to infectious disease modelling.

## Methods

### Search strategy and article selection

We conducted searches on Medline (via Ovid) [11], Web of Science [12], medRxiv [13], and bioRxiv [14] on January 4th, 2024 to find articles that provide guidance on writing or assessing modelling studies. The search was not restricted temporally or by language and was developed in collaboration with an information specialist. Further details about these searches, including the search strings used and the number of hits for each search, are given in subsection 1.1 of Additional File 1. Although the focus of the review was on infectious disease modelling, we included search terms to find guidelines for modelling studies in other fields—especially health economics—since some of the fundamental reporting and assessment tools for these types of models may also be applicable to infectious disease models.

The articles found by these searches were aggregated and uploaded into Covidence and, after the removal of duplicates, filtered through double screening [15]. Screening was conducted in two rounds—first a title-and-abstract screening (AB, MC, VKJ), and then a full-text screening (AB, MC, JS). Conflicts at both screening stages were resolved by discussion (AB, MC, JS, VKJ). We included published articles that made original recommendations for the reporting or quality assessment of dynamic or decision-analytic modelling studies. Articles that made recommendations or presented guidelines for clinical course models and specific drug or treatment assessments were excluded, as were articles which made recommendations that were deemed irrelevant or non-generalisable to infectious disease dynamic or decision-analytic modelling (e.g. recommendations for structural equation modelling, guidelines for conducting systematic reviews, etc.). When articles were reviews of quality assessment tools, reporting guidelines, or best practice recommendations in themselves, they were only included if the authors made suggestions or recommendations of their own that went further than the recommendations made by the articles included in the review. The individual articles included in such a review were also considered for inclusion in our study. The complete and exact inclusion and exclusion criteria for the screening rounds are given in subsubsection 1.1.3 of Additional File 1. A full protocol for the scoping review is available on the Open Science Framework platform [16].

### Identification of articles via other methods

In an effort to include articles beyond the scope of our search strategy, we considered additional methods of article identification. We included guideline articles known to us or brought to our attention that had not appeared in the search, as well as relevant articles found by snowballing (also done for articles we eventually excluded). Additionally, we searched the EQUATOR (Enhancing the QUAlity and Transparency Of health Research) Network library [17] to determine whether any reporting guidelines for infectious disease modelling studies were published there or were reported to be under development. A search of a random subset of 100 recently published infectious disease modelling studies was also performed to see whether any studies reported following guidelines (see Sect. 1.2 of Additional File 1 for more details). Identified guidelines which followed our inclusion and exclusion criteria, as defined in Sect. 1.1.3 of Additional File 1, were included.

### Data extraction and analysis

Article extraction was conducted by AB and MC. The main information that we extracted was individual recommendations (e.g. one bullet point in a list, one question in a checklist, etc.) made by the included articles; we will henceforth refer to an extracted recommendation as an “item”. When the only recommendation an article made concerning a specific topic was a reference to another guideline that should be followed, this was not deemed eligible for extraction. Recommendations deemed irrelevant to infectious disease modelling studies (e.g. recommendations about how to consider or calculate costs during a health economic analysis) by the extractors were not extracted. Metadata like the title, year of publication, and doi of included articles were also manually extracted, as were additional details such as the field of research an article was concerned with, and whether it was itself a literature review.

To facilitate the identification of common themes in recommendations across the included articles, items were classified into categories—henceforth known as subdimensions—defined by thematic similarities. The initial number and definition of these subdimensions was determined by grouping together recommendations made by two quality assessment tools—Caro 2014, a published checklist intended for use by the scientific community to assess the credibility and relevance of modelling studies to inform healthcare decision-making [18], and a bespoke checklist created specifically to assess the quality of modelling studies included in a rapid review (Burns 2021) of travel-related control measures for SARS-CoV-2 [19]. We chose specifically these two tools as the starting point since we had previously used them to assess the quality of several modelling studies to inform policy-makers during the COVID-19 pandemic and were thus already familiar with their recommendations. The examples below demonstrate how these two tools were used to initially define subdimensions:

- One item in Caro 2014 [18] reads “Is internal verification of the model sufficient to make its results credible for your decision?”. The checklist from Burns 2021 [19] asks a related question— “Has an internal validation process been described? Has the model been shown to be internally valid?”. Therefore, we created a subdimension that would include items about the internal validity of modelling studies.
- The checklist in Burns 2021 [19] does not require an assessment of the face validity of a modelling study, but Caro 2014 [18] instructs the assessor to answer the question “Does the model have sufficient face validity to make its results credible for your decision?”. Therefore, we created a subdimension to include items about the face validity of modelling studies. In this example, only Caro 2014 would be initially considered as addressing this subdimension.

When items extracted from subsequent articles did not thematically fit into an existing subdimension, a new subdimension was created (for example, neither Caro 2014 nor the checklist from Burns 2021 addressed model calibration, so the extraction of the first article to mention calibration resulted in the creation of a subdimension for calibration). Once all guidelines had been extracted, the list of subdimensions was finalised, and we reviewed the classification of items to check whether some items would fit better into a different subdimension that may only have been created after the initial classification of that item. These subdimensions were sorted into overarching groups based on similarities; these overarching groups are denoted as “dimensions” throughout the manuscript. An article was considered as having mentioned a dimension if it included at least one of the subdimensions belonging to that dimension. Articles which were very narrowly focused (i.e. only covered one dimension) were excluded from the final analysis.

Furthermore, to enable a differentiated analysis of (sub-)dimension prevalence depending on the intended use of the extracted articles, we classified each article as a reporting guideline or a quality assessment tool. These categories were not mutually exclusive; some articles gave best practice recommendations, which included aspects of both reporting and quality assessment. These articles were classified as both a reporting guideline and a quality assessment tool.

Most articles were extracted by a single extractor. However, 20 articles (38%) were extracted independently by both extractors to validate the extraction process. Articles which were not available in English or German were translated using DeepL [20]. The data extraction sheet can be found in the Git repository linked in the data availability statement.

All analyses of the extracted information were performed using R version 4.4.2 [21], and all figures in the manuscript were created using the R package ggplot version 3.5.1 [22]. We adhered to the PRISMA checklist extension for scoping reviews throughout this study [23] (Additional File 2).

## Results

**Fig. 1:**
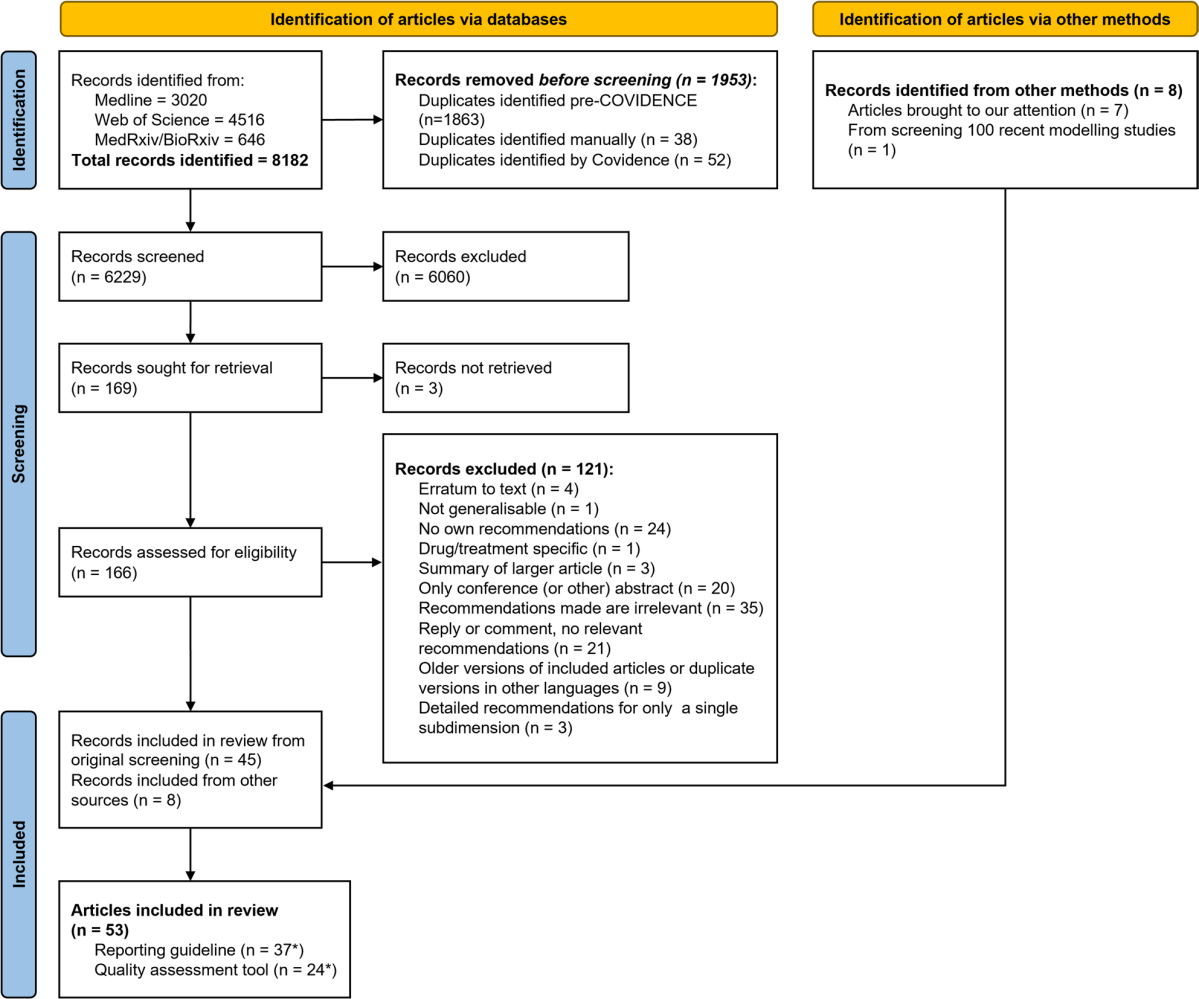
PRISMA flowchart of articles included in the scoping review. For more details on the search, please see Sect. 1.1 of Additional file 1. *Eight articles were classified as being applicable both as a reporting guideline and as a quality assessment tool; therefore, the number of reporting guidelines (*n* = 37) and quality assessment tools (*n* = 24) adds up to more than the 53 identified articles

Details about the number of articles that progressed through each round of screening can be found in Fig. 1. We identified 8182 articles from the queried databases. After title-and-abstract and full-text screening, we were left with 45 articles. We supplemented these with eight articles that were known to us or were brought to our attention that had not appeared in our search [19, 24–30], leading to a total of 53 articles eligible for extraction [3, 18, 19, 24–73]. Of these, 11 (21%) were reviews of quality assessment tools, reporting guidelines, or best practice recommendations [3, 26, 31, 35, 42, 55, 60, 66, 69, 71, 73], but added their own recommendations.

### Type of guideline

The identified articles included best practice model development guidelines, reporting recommendations, and quality assessment tools. Initially, 29 articles (55%) were classified as reporting guidelines [3, 24–29, 31–34, 36, 38, 40, 41, 46, 48, 51, 54–56, 61, 62, 64, 65, 67, 71–73] and 16 articles (30%) fell into the category of quality assessment tools [18, 19, 35, 42–45, 47, 49, 52, 53, 59, 60, 63, 66, 68]. Eight articles (15%) presented best practice recommendations, which included aspects of model development and reporting and can also be used to assess the quality of studies [30, 37, 39, 50, 57, 58, 69, 70]; therefore, we grouped these articles into both the reporting guideline and the quality assessment tool categories. Therefore, we ended up with a total of 37 reporting guidelines and 24 quality assessment tools despite only having 53 unique articles.

### Modelling field

Of the 53 guidelines, 33 (62%) were concerned with models or modelling studies for health economic evaluations (Additional file 1 Figure S1.3.1a) [26, 28, 31–34, 38, 39, 42–55, 57, 59–61, 64–66, 69, 71–73]. Eleven of the 53 articles (21%) were about infectious disease models [24, 30, 35–37, 41, 62, 63, 67, 68, 70], but they were all narrow in their scope, being restricted in terms of the type(s) of pathogens being modelled or the intended use of the models. For example, two of these 11 focused specifically on modelling drug-resistant infections in healthcare or long-term care settings [63, 67], and a further two discussed models specifically designed for epidemic forecasting and analysing emerging outbreaks [36, 70]. Nine of the 53 articles (17%) fell into a field other than health economics or infectious disease modelling: seven articles (13%) considered a wider scope of models used to answer healthcare questions without being restricted to the health economic perspective or limited to infectious disease models [3, 18, 19, 27, 40, 56, 58] and two articles were not explicitly focused on healthcare—one described a protocol for reporting agent-based models [29] and the other gave recommendations for describing simulation models regardless of type or purpose [25]. For insight into how recommendations varied by modelling field and which were most prevalent in infectious disease modelling guidelines, please refer to Sect. 1.3.1 of Additional File 1.

### Dimensions and subdimensions

**Fig. 2:**
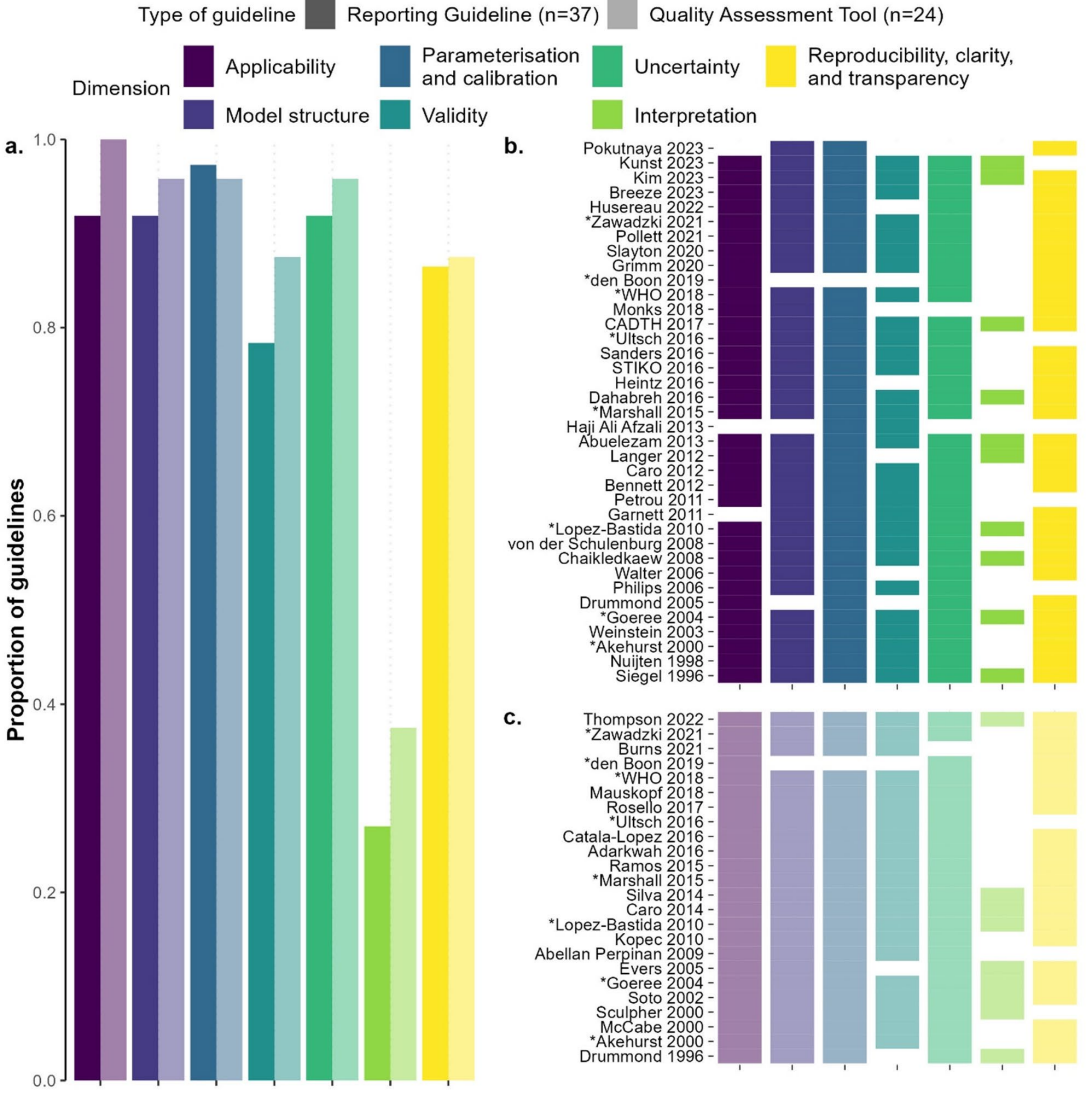
Prevalence of each dimension in the reviewed guidelines. (**a**) Overall proportion of guidelines mentioning each dimension, stratified by type of guideline. (**b**) Presence or absence of each dimension by reporting guideline. (**c**) Presence or absence of each dimension by quality assessment tool. The data used for these plots can be found in the Git repository linked in the data availability statement. A tabular overview of the information presented here can be found in Sect. 1.3.3 of Additional File 1. *Eight guidelines are part of both reporting guidelines and quality assessment tools since both aspects are covered in the guidelines

**Fig. 3:**
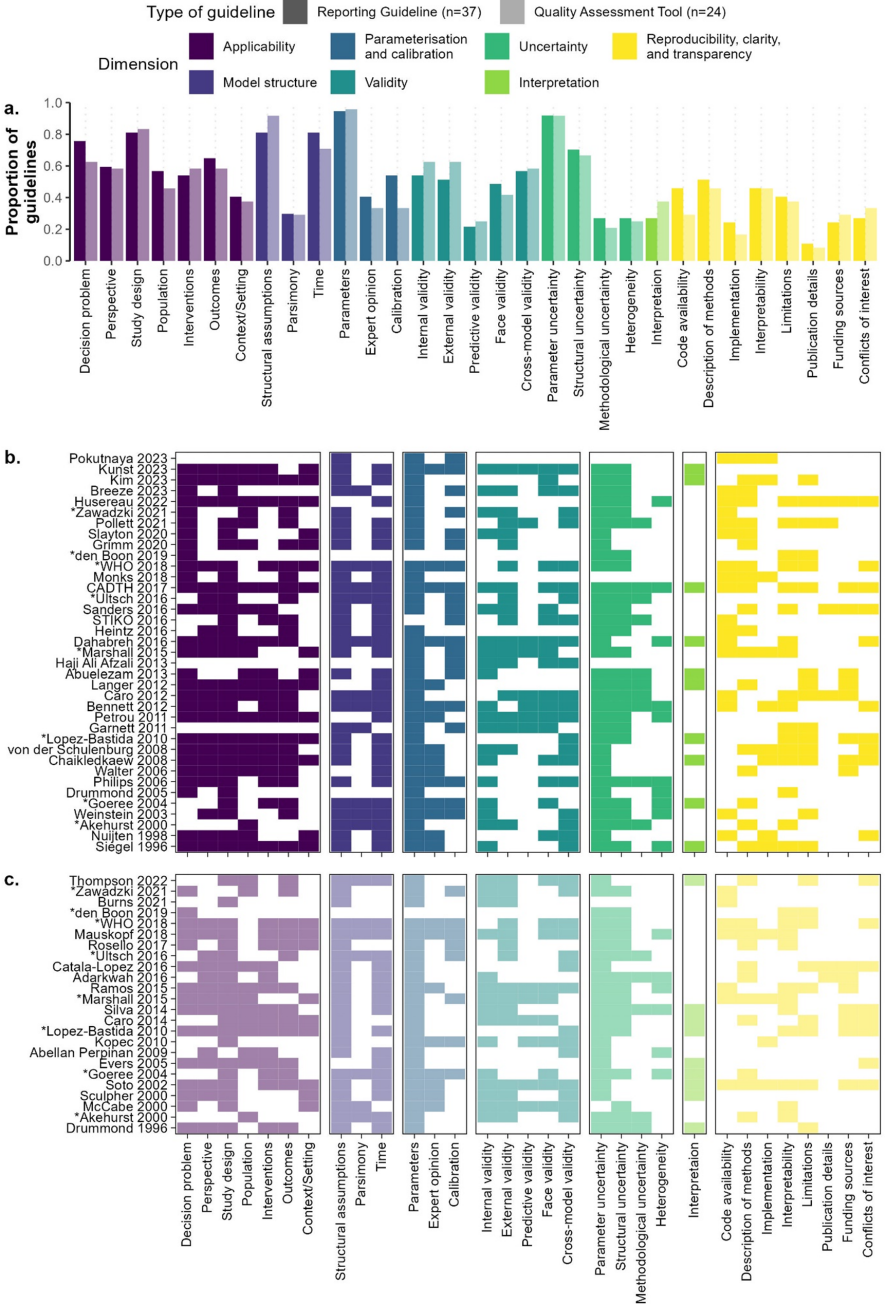
Prevalence of each subdimension in the reviewed guidelines. (**a**) Overall proportion of guidelines mentioning each subdimension, stratified by type of guideline. (**b**) Presence or absence of each subdimension by reporting guideline. (**c**) Presence or absence of each subdimension by quality assessment tool. The data used for these plots can be found in the Git repository linked in the data availability statement. A tabular overview of the information presented here can be found in Sect. 1.3.3 of Additional File 1. *Eight guidelines are part of both reporting guidelines and quality assessment tools since both aspects are covered in the guidelines

We classified recommendations from the selected articles into 31 subdimensions which were then grouped into seven broad dimensions, each representing a different facet of a modelling study (Table 1; Figs. 2 and 3). These seven dimensions were: *applicability*; *model structure*; *parameterisation and calibration*; *validity*; *uncertainty*; *interpretation*; and *reproducibility*,* clarity*,* and transparency*. For a detailed overview of what each dimension and subdimension entails, please refer to Sect. 1.3.2 of Additional file 1.

Guidelines covered a median of 6 dimensions and 16 subdimensions. Eight reporting guidelines (22%) [26, 28, 39, 41, 46, 48, 57, 65] and six quality assessment tools (25%) [18, 39, 44, 57, 66, 68] covered all seven dimensions (Fig. 2b, c). Furthermore, we observed no clear temporal trend in the frequency with which guidelines cover specific dimensions or subdimensions (Figs. 2b and c and 3b and c).

**Table 1:** Overview of dimensions and subdimensions

Dimension/Subdimension	Assessment/Reporting criteria
***1. Applicability***	*Considers the background/basis of the modelling study*
1.1 Decision problem	Is there a clear statement of the aim/decision problem?
1.2 Perspective	Is the perspective (i.e. scope (e.g. societal or a specific economics/healthcare system) considered in the model) of the study clearly stated and/or relevant?
1.3 Study design	Is the study design clearly stated and/or applicable to the decision problem?
1.4 Population	Is the population clearly stated and/or applicable to the decision problem?
1.5 Interventions	Is there a comprehensive consideration of the interventions and/or comparators?
1.6 Outcomes	Are the outcomes clearly stated and/or applicable to the decision problem?
1.7 Context/Setting	Is the context/setting clearly stated and/or applicable to the decision problem?
***2. Model structure***	*Considers key elements of model structure*,* including structural assumptions*,* parsimony*,* and time*
2.1 Structural assumptions	Are structural and methodological assumptions clearly stated and reasonable?
2.2 Parsimony	Is the model as simple as possible but as complex as necessary?
2.3 Time	Is the time horizon and/or time step clearly stated and appropriate?
***3. Parameterisation and calibration***	*Considers elements of parameterisation and calibration of the model*
3.1 Parameters	Are parameter values transparent and reasonable? Are data sources and their translation into parameter values transparent and justified?
3.2 Expert opinion	If applicable, has the methodology used to elicit expert opinion for parameterisation been sufficiently described?
3.3 Calibration	If applicable, were methods and data sources used for calibration described in sufficient detail? Were the subsequent results presented clearly?
***4. Validity***	*Considers key types of model validation*
4.1 Internal validity	Has evidence of the mathematical soundness and correctness of model implementation been given?
4.2 External validity	Has the model been validated against independent data sources?
4.3 Predictive validity	Has the ability of the model to predict future events been shown?
4.4 Face validity	Is the model structure plausible? Have any counterintuitive results been explained?
4.5 Cross-model validity	Has the model been validated against similar models?
***5. Uncertainty***	*Considers different types of uncertainty found in the model and in model results*
5.1 Parameter uncertainty	Have the methods used to assess uncertainty surrounding parameters been clearly described?
5.2 Structural uncertainty	Have the methods used to assess uncertainty arising from structural assumptions been clearly described?
5.3 Methodological uncertainty	Have the methods used to assess uncertainty stemming from the choice of methodology been clearly described?
5.4 Heterogeneity	Have the methods used to assess variability in results across subgroups been clearly described?
***6. Interpretation***	*Considers how the authors interpret the results of the model*
6.1 Interpretation	Is the presented interpretation of the results reasonable, fair, and/or balanced?
***7. Reproducibility***,*** clarity***,*** and transparency***	*Considers key elements to ensure a modelling study is reproducible and transparently reported*
7.1 Code availability	Are the code/model/data available?
7.2 Description of methods	Are methods described transparently and in enough detail that they can be reproduced?
7.3 Implementation	Has the choice of software been stated and justified?
7.4 Interpretability	Is there sufficient non-technical documentation and description?
7.5 Limitations	Are limitations clearly described?
7.6 Publication details	Is the type of study clearly identified in the title or abstract? Has the model developer been stated?
7.7 Funding sources	Are the funding sources and the role of the funder in the study clearly stated?
7.8 Conflicts of interest	Are there any potential conflicts of interest?

List of dimensions (in ***bold italics***) and subdimensions as well as a brief summarised question that addresses the elements mentioned by guidelines (for reporting as well as for quality assessment) that were extracted and classified into each subdimension. The dimensions and subdimensions reflect the elements covered by the included guidelines and are not a judgement of what we believe a future infectious disease modelling guideline should include

*Applicability* was covered by 34 of the 37 reporting guidelines (92%; Fig. 2) and all 24 quality assessment tools. The subdimension *study design*, containing items about the appropriateness of the study design given the research question, was the most well-represented subdimension under this dimension, with 30 reporting guidelines (81%; Fig. 3) and 20 quality assessment tools (83%) mentioning items that were classified into this subdimension.

The dimension *model structure* was also covered by 34 of the 37 reporting guidelines (92%) and 23 of the 24 quality assessment tools (96%). Two of the three subdimensions in this dimension were mentioned by more than 70% of the articles; *structural assumptions*, which contained items about the reporting or quality assessment of the structural assumptions made by a model, was covered by 30 reporting guidelines (81%) and 22 quality assessment tools (92%) and *time*, which included items about the cycle length and time horizon of the model, was covered by 30 reporting guidelines (81%) and 17 quality assessment tools (71%).

*Parameterisation and calibration* was the most prevalent dimension for reporting guidelines (36 of 37 studies, 97%) and one of the second most frequently mentioned dimensions for quality assessment tools, being covered in 23 of 24 such guidelines (96%). The only guideline which did not cover this dimension focused specifically on multi-model comparisons and served as both a reporting guideline and a quality assessment tool [37]. This dimension was most frequently represented by the subdimension *parameters* for both reporting guidelines (*n* = 35, 95%) and quality assessment tools (*n* = 23, 96%). This subdimension contained items about the transparency and/or validity of the parameter values used in an infectious disease model as well as the primary data sources used to elicit them. The only article aside from den Boon 2017 [37] that did not cover the *parameters* subdimension was STIKO 2016 [24], which only mentions in passing that “[r]elevant guidelines must be followed especially when using secondary data” and refers to other guidelines.

The dimension *validity* contained items about reporting or assessing the process and results of model validation in a modelling study and was covered by 29 reporting guidelines (78%) and 21 quality assessment tools (88%). The subdimensions in this dimension represent the different types of validity a model may possess. The most frequently mentioned of these were *internal validity*, comprising items about the soundness of the mathematical logic of the model, absence of bugs in code implementation, which appeared in 20 reporting guidelines (54%) and 15 quality assessment tools (62%), *cross-model validity*, which consisted of items discussing validating a model against other models or modelling studies and was mentioned by 21 reporting guidelines (57%) and 14 quality assessment tools (58%), and *external validity*, which covered items on validation against independent datasets and was mentioned by 19 reporting guidelines (51%) and 15 quality assessment tools (62%).

*Uncertainty* was covered by 34 of the 37 reporting guidelines (92%) and 23 of the 24 quality assessment tools (96%). This dimension was split into five subdimensions corresponding to five types of uncertainty in a modelling study; of these, the subdimension *parameter uncertainty*, containing items about how modelling studies dealt with uncertainty around parameter values, was the most frequently mentioned, having been covered by 34 reporting guidelines (92%) and 22 quality assessment tools (92%).

*Interpretation* was the least prevalent dimension. Containing only one eponymous subdimension, it included items that discussed the fairness and reasonableness of the authors’ interpretation of the results of a modelling study. This (sub-)dimension was only covered by 10 reporting guidelines (27%) and nine quality assessment tools (38%).

The dimension *reproducibility*,* clarity*,* and transparency* comprised items aimed at making modelling studies easier to reproduce and understand for the layperson, as well as items about funding sources and conflicts of interest. Items that were classified into this dimension were present in 32 of the 37 reporting guidelines (86%) and 21 of the 24 quality assessment tools (88%). None of the subdimensions for this dimension were mentioned by a majority of all guidelines, but *description of methods*—which comprised items about the extent to which the methods of a modelling study were described and whether they were completely reproducible—came the closest, having been mentioned by 19 reporting guidelines (51%) and 11 quality assessment tools (46%).

## Discussion

With this study, we aimed to identify common dimensions and subdimensions appearing in guidelines for modelling studies by assessing reporting guidelines and quality assessment tools from infectious disease modelling and neighbouring fields. We found 53 relevant guidelines from which a variety of common dimensions and subdimensions were identified. The identified dimensions and subdimensions reflect the elements covered by the included guidelines and are not a direct judgement of what we believe a future infectious disease modelling guideline should include.

The transparent reporting of parameter values and their sources was the most frequently mentioned subdimension across all studies. A clear description of the assessment of uncertainty around parameters and justification of the structural assumptions were also prominent subdimensions. Having a well-described study design that is appropriate for the decision problem was also recommended by the vast majority of articles. These subdimensions could form a starting point for the next step of developing a minimal set of core items for inclusion in a guideline for infectious disease modelling. This set is however by no means exhaustive. Many subdimensions appearing less frequently are arguably equally important; this includes, for example, those touching on reporting conflicts of interest, the validation of models before use, or the interpretability of the study by a non-technical audience (especially policy makers). The less frequently mentioned elements which are still important could provide insight into which areas may require extra attention to ensure better transparency in reporting and more accurate quality assessment.

One potential limitation of this study is the fact that several guidelines were identified through personal communication rather than through our search strategy, suggesting that our search strategy may not have been broad enough to identify all relevant guidelines. Consequently, there may still be some guidelines which have not been assessed in this review, though snowballing was used to minimise this possibility. However, this difficulty in identifying relevant guidelines (e.g. because of wide variations in terminology and differing levels of specificity in naming) could also be part of the reason why reporting guidelines do not currently appear to be used frequently for infectious disease modelling studies.

It is also important to consider that the subdimensions may have varying applicability and relevance to infectious disease modelling. For example, structural assumptions and parameter values are crucial to an infectious disease model—as with any other model—and the corresponding subdimensions were addressed by a majority of the articles concerned with infectious disease modelling. On the other hand, establishing predictive validity may be impossible in some situations where infectious disease models are needed, and a choice of perspective is much more relevant to health economic evaluations than infectious disease models. Thus, when applying the findings of these guidelines to recommendations for infectious disease models, it is important to keep the diversity of fields covered in this review in mind. Given that the majority of included guidelines came from the field of health economics, the overall results may also be skewed slightly in favour of recommendations relevant for health economics even if they are not as important for infectious disease modelling; a closer look at how recommendation prevalence differed between fields can be found in Additional File 1 Sect. 1.3.1.

The definitions of the various subdimensions in our study are subjective, as are the classifications of recommendations into these subdimensions. This means that some subdimensions are broader than others, and that elements covered in individual articles could be classified into several different subdimensions. This would impact the frequency with which certain subdimensions are mentioned. Furthermore, due to the fact that the structural starting point of our categorisation was based on the recommendations and structure of two already existing quality assessment tools, our own classifications may reinforce existing biases already present in those two tools. This is exacerbated by the fact that judgements on relevance of recommendations made in the reviewed guidelines were made based on the subjective opinions of the extractors, albeit after discussion to introduce some objectivity. Consequently, the results are, to some extent, an oversimplification of the true complexity and diversity of the articles used in this study. A deeper look at the content of individual guidelines may be helpful to better understand the depth and intricacies of existing reporting guidelines and quality assessment tools. Nonetheless, our results provide an overview of the numerous dimensions and subdimensions that commonly occur in reporting guidelines and quality assessment tools for modelling studies.

It is possible that the field of infectious disease modelling as a whole is too broad to benefit from generalised reporting guidelines and quality assessment tools. Even if this is the case, we believe that an overall assessment of recommendations currently in place for infectious disease modelling and neighbouring fields, as done in this review, provides valuable insight into common recommendations and indicators of model quality regardless of the specific focus of a modelling study. Thus, the results presented here could be used to create specialised guidelines that share a common root, thus also allowing for comparisons of modelling studies of different types. The analysis of how often certain recommendations are mentioned can also provide a guide to valuable recommendations that have been neglected so far, and where more focus needs to be laid in future guidelines.

## Conclusions

This review demonstrates the existence of many common topics and recommendations towards standardised guidelines for modelling studies. The more common use of infectious disease modelling in the context of the COVID-19 pandemic supports the need of defining such specified guidelines. To this end, the common recommendations we have identified in this review of guidelines from specific areas of infectious disease modelling and neighbouring fields could be adapted to create guidelines for infectious disease modelling as a whole or to develop a suite of tools aimed at different types of infectious disease modelling studies. Establishing such recommendations could be monumental in assisting researchers and policy makers by facilitating the inclusion of modelling studies in the public health decision-making process. Therefore, as a follow-up to this review, our next steps will be to develop a quality assessment tool and a reporting guideline by building upon the subdimensions identified in this review.

## Supplementary Information

Below is the link to the electronic supplementary material.

Supplementary Material 1: Additional File 1: “Search strategy and additional results”. This file contains details on the search strategy as well as an analysis of results stratified by field (health economics, infectious diseases, and other).

Supplementary Material 2: Additional File 2: “Preferred Reporting Items for Systematic reviews and Meta-Analyses extension for Scoping Reviews (PRISMA-ScR) Checklist” containing our PRISMA-ScR Checklist.

## Data Availability

The datasets generated and/or analysed during the current study are available in the GIT repository, https://zivgitlab.uni-muenster.de/clinical-epi/ReportingGuidelines.

## Abbreviations

CADTH: Canadian Agency for Drugs and Technologies in Health
EQUATOR: Enhancing the QUAlity and Transparency Of health Research
STIKO: German Standing Committee on Vaccination
WHO: World Health Organisation

## References

[1] Ferrari AJ, Santomauro DF, Aali A, Abate YH, Abbafati C, Abbastabar H, et al. Global incidence, prevalence, years lived with disability (YLDs), disability-adjusted life-years (DALYs), and healthy life expectancy (HALE) for 371 diseases and injuries in 204 countries and territories and 811 subnational locations, 1990–2021: a systematic analysis for the global burden of disease study 2021. Lancet. 2024;403(10440):2133–61. 38642570 10.1016/S0140-6736(24)00757-8 PMC11122111

[2] McBryde ES, Meehan MT, Adegboye OA, Adekunle AI, Caldwell JM, Pak A, et al. Role of modelling in COVID-19 policy development. Paediatr Respir Rev. 2020;35:57–60. 32690354 10.1016/j.prrv.2020.06.013 PMC7301791

[3] Bennett C, Manuel DG. Reporting guidelines for modelling studies. BMC Med Res Methodol [Internet]. 2012 Nov 7 [cited 2024 Jun 12];12(1):1–7. Available from: https://bmcmedresmethodol.biomedcentral.com/articles/10.1186/1471-2288-12-168. 10.1186/1471-2288-12-168 PMC3533955 23134698

[4] Barnett B, Townley L, Post V, Evans R, Hunt R, Peeters L, et al. Australian groundwater modelling guidelines. Canberra: National Water Commission [Internet]. 2012. Available from: http://archive.nwc.gov.au/library/waterlines/82.

[5] Grimm V, Berger U, DeAngelis DL, Polhill JG, Giske J, Railsback SF. The ODD protocol: a review and first update. Ecol Modell. 2010;221(23):2760–8.

[6] The lancet commission on strengthening the use of epidemiological modelling of emerging and pandemic infectious diseases. How modelling can better support public health policy making: the lancet commission on strengthening the use of epidemiological modelling of emerging and pandemic infectious diseases. The Lancet [Internet]. 2024 [cited 2025 May 21];403(10429):789–91. Available from: https://www.thelancet.com/journals/lancet/article/PIIS0140-6736(23)02758-7/fulltext. 10.1016/S0140-6736(23)02758-7 38141627

[7] EQUATOR Network |. Enhancing the QUAlity and Transparency Of Health Research [Internet]. [cited 2023 Nov 6]. Available from: https://www.equator-network.org/.

[8] von Elm E, Altman DG, Egger M, Pocock SJ, Gøtzsche PC, Vandenbroucke JP. The strengthening the reporting of observational studies in epidemiology (STROBE) statement: guidelines for reporting observational studies. J Clin Epidemiol. 2008;61(4):344–9. 18313558 10.1016/j.jclinepi.2007.11.008

[9] Moher D, Liberati A, Tetzlaff J, Altman DG. Preferred reporting items for systematic reviews and meta-analyses: the PRISMA statement. BMJ [Internet]. 2009 Jul 21 [cited 2023 Nov 6];339(7716):332–6. Available from: https://www.bmj.com/content/339/bmj.b2535. PMC3090117 21603045

[10] Higgins JPT, Altman DG, Gøtzsche PC, Jüni P, Moher D, Oxman AD, et al. The cochrane collaboration’s tool for assessing risk of bias in randomised trials. BMJ [Internet]. 2011 Oct 29 [cited 2023 Nov 6];343(7829). Available from: https://pubmed.ncbi.nlm.nih.gov/22008217/. 10.1136/bmj.d5928 PMC3196245 22008217

[11] Ovid - Ovid. MEDLINE^®^ | Wolters Kluwer [Internet]. [cited 2024 Jun 20]. Available from: https://www.wolterskluwer.com/en/solutions/ovid/ovid-medline-901.

[12] Advanced search - Web of Science Core Collection [Internet]. [cited 2024 Jun 20]. Available from: https://www.webofscience.com/wos/woscc/advanced-search.

[13] medRxiv.org. - the preprint server for Health Sciences [Internet]. [cited 2024 Jun 20]. Available from: https://www.medrxiv.org/.

[14] bioRxiv.org. - the preprint server for Biology [Internet]. [cited 2024 Jun 20]. Available from: https://www.biorxiv.org/.

[15] Covidence systematic review software [Internet]. Veritas Health Innovation, Melbourne, Australia; Available from: https://www.covidence.org.

[16] Chaturvedi M, Bartz A, Denkinger CM, Klett-Tammen C, Kretzschmar M, Kuhlmann A, et al. Reporting and quality assessment guidelines for infectious disease modelling studies: a scoping review. 2024 Feb 9 [cited 2024 Jun 20]; Available from: https://osf.io/ab6d3.

[17] EQUATOR Network |. Enhancing the QUAlity and Transparency Of Health Research [Internet]. [cited 2024 Jun 20]. Available from: https://www.equator-network.org/.

[18] Caro JJ, Eddy DM, Kan H, Kaltz C, Patel B, Eldessouki R, et al. Questionnaire to assess relevance and credibility of modeling studies for informing health care decision making: an ISPOR-AMCP-NPC good practice task force report. Value Health [Internet]. 2014 [cited 2024 Jun 12];17(2):174–82. Available from: https://pubmed.ncbi.nlm.nih.gov/24636375/. 10.1016/j.jval.2014.01.003 24636375

[19] Burns J, Movsisyan A, Stratil JM, Biallas RL, Coenen M, Emmert-Fees KMF et al. International travel-related control measures to contain the COVID-19 pandemic: a rapid review. Cochrane Database Syst Rev. 2021;2021(3). 10.1002/14651858.CD013717.pub2 PMC8406796 33763851

[20] DeepL SE. DeepL [Internet]. [cited 2024 Jul 10]. Available from: https://www.deepl.com/en/translator/files.

[21] R Core Team. R: A language and environment for statistical computing [Internet]. Vienna, Austria. 2024. Available from: https://www.R-project.org/.

[22] Wickham H. ggplot2: Elegant graphics for data analysis. Springer-Verlag New York [Internet]. 2016. Available from: https://ggplot2.tidyverse.org.

[23] Tricco AC, Lillie E, Zarin W, O’Brien KK, Colquhoun H, Levac D, et al. PRISMA extension for scoping reviews (PRISMA-ScR): Checklist and explanation. Ann Intern Med [Internet]. 2018 Oct 2 [cited 2024 Jun 20];169(7):467–73. Available from: 10.7326/M18-0850. https://www.acpjournals.org. 10.7326/M18-0850 30178033

[24] STIKO. Modelling methods for predicting epidemiological and health economic effects of vaccinations. Guidance for analyses to be presented to the German Standing Committee on Vaccination (STIKO) [Internet]. Berlin; 2016 Mar [cited 2024 Jan 2]. Available from: https://www.nitag-resource.org/sites/default/files/fcf579b0fd106551e22cbebabb28c56ae0a058e2_1.pdf.

[25] Monks T, Currie CSM, Onggo BS, Robinson S, Kunc M, Taylor SJE. Strengthening the reporting of empirical simulation studies: introducing the STRESS guidelines. J Simul [Internet]. 2018 Jan 2 [cited 2024 Jun 12];13(1):55–67. Available from: https://www.tandfonline.com/doi/abs/10.1080/17477778.2018.1442155.

[26] Kim DD, Do LA, Synnott PG, Lavelle TA, Prosser LA, Wong JB, et al. Developing criteria for health economic quality evaluation tool. Value Health. 2023;26(8):1225–34. 37068557 10.1016/j.jval.2023.04.004

[27] Garnett GP, Cousens S, Hallett TB, Steketee R, Walker N. Mathematical models in the evaluation of health programmes. Lancet [Internet]. 2011 [cited 2024 Jul 12];378(9790):515–25. Available from: https://pubmed.ncbi.nlm.nih.gov/21481448/. 10.1016/S0140-6736(10)61505-X 21481448

[28] CADTH. Guidelines for the economic evaluation of health technologies: Canada. 4th ed. [Internet]. Ottawa; 2017 Mar [cited 2024 Jun 12]. Available from: https://www.cadth.ca/guidelines-economic-evaluation-health-technologies-canada-0.

[29] Grimm V, Railsback SF, Vincenot CE, Berger U, Gallagher C, DeAngelis DL, et al. The ODD protocol for describing agent-based and other simulation models: a second update to improve clarity, replication, and structural realism. J Artif Soc Soc Simul [Internet]. 2020 [cited 2024 Dec 4];23(2). Available from: http://jasss.soc.surrey.ac.uk/23/2/7.html.

[30] WHO. Guidance for country-level TB modelling [Internet]. 2018 [cited 2025 Jan 30]. Available from: https://www.who.int/publications/i/item/9789241514521.

[31] Philips Z, Bojke L, Sculpher M, Claxton K, Golder S. Good practice guidelines for decision-analytic modelling in health technology assessment: a review and consolidation of quality assessment. Pharmacoeconomics [Internet]. 2006 Oct 9 [cited 2024 Jun 12];24(4):355–71. Available from: https://link.springer.com/article/10.2165/00019053-200624040-00006. 10.2165/00019053-200624040-00006 16605282

[32] Weinstein MC, O’Brien B, Hornberger J, Jackson J, Johannesson M, McCabe C, et al. Principles of good practice for decision analytic modeling in Health-Care evaluation: report of the ISPOR task force on good research practices—Modeling studies. Value Health. 2003;6(1):9–17. 12535234 10.1046/j.1524-4733.2003.00234.x

[33] Nuijten MJC, Pronk MH, Brorens MJA, Hekster YA, Lockefeer JHM, De Smet PAGM, et al. Reporting format for economic evaluation. Part II: Focus on modelling studies. Pharmacoeconomics [Internet]. 1998 [cited 2024 Jun 12];14(3):259–68. Available from: https://pubmed.ncbi.nlm.nih.gov/10186465/. 10.2165/00019053-199814030-00003 10186465

[34] Drummond M, Manca A, Sculpher M. Increasing the generalizability of economic evaluations: recommendations for the design, analysis, and reporting of studies. Int J Technol Assess Health Care [Internet]. 2005 [cited 2024 Jun 12];21(2):165–71. Available from: https://www.cambridge.org/core/journals/international-journal-of-technology-assessment-in-health-care/article/increasing-the-generalizability-of-economic-evaluations-recommendations-for-the-design-analysis-and-reporting-of-studies/A0339C7616242B177244D80B73031B2B. 15921055

[35] Kopec JA, Finès P, Manuel DG, Buckeridge DL, Flanagan WM, Oderkirk J, et al. Validation of population-based disease simulation models: a review of concepts and methods. BMC Public Health [Internet]. 2010 Nov 18 [cited 2024 Jun 12];10(1):1–13. Available from: 10.1186/1471-2458-10-710. https://bmcpublichealth.biomedcentral.com/articles/. 10.1186/1471-2458-10-710 PMC3001435 21087466

[36] Pollett S, Johansson MA, Reich NG, Brett-Major D, Del Valle SY, Venkatramanan S, et al. Recommended reporting items for epidemic forecasting and prediction research: The EPIFORGE 2020 guidelines. PLoS Med [Internet]. 2021 Oct 1 [cited 2024 Jun 12];18(10). Available from: https://pubmed.ncbi.nlm.nih.gov/34665805/. 10.1371/journal.pmed.1003793 PMC8525759 34665805

[37] Den Boon S, Jit M, Brisson M, Medley G, Beutels P, White R, et al. Guidelines for multi-model comparisons of the impact of infectious disease interventions. BMC Med [Internet]. 2019 Aug 19 [cited 2024 Jun 12];17(1):1–13. Available from: 10.1186/s12916-019-1403-9. https://bmcmedicine.biomedcentral.com/articles/. 10.1186/s12916-019-1403-9 PMC6699075 31422772

[38] Husereau D, Drummond M, Augustovski F, de Bekker-Grob E, Briggs AH, Carswell C, et al. Consolidated Health Economic Evaluation Reporting Standards 2022 (CHEERS 2022) statement: updated reporting guidance for health economic evaluations. BMC Med [Internet]. 2022 Dec 1 [cited 2024 Jun 12];20(1):1–8. Available from: 10.1186/s12916-021-02204-0. https://bmcmedicine.biomedcentral.com/articles/. 10.1186/s12916-021-02204-0 PMC8753858 35022047

[39] Goeree R, O’Brien BJ, Blackhouse G. Principles of good modeling practice in healthcare cost-effectiveness studies. Expert Rev Pharmacoecon Outcomes Res. 2004;4(2):189–98. 19807523 10.1586/14737167.4.2.189

[40] Caro JJ, Briggs AH, Siebert U, Kuntz KM. Modeling good research practices—Overview: A report of the ISPOR-SMDM modeling good research practices task Force-1. Value Health. 2012;15(6):796–803. 22999128 10.1016/j.jval.2012.06.012

[41] Abuelezam NN, Rough K, Seage GR. Individual-based simulation models of HIV transmission: reporting quality and recommendations. PLoS One [Internet]. 2013 Sep 30 [cited 2024 Jun 11];8(9):e75624. Available from: https://journals.plos.org/plosone/article?id=10.1371/journal.pone.0075624. 10.1371/journal.pone.0075624 PMC3787035 24098707

[42] Peñaloza Ramos MC, Barton P, Jowett S, Sutton AJ. A systematic review of research guidelines in decision-analytic modeling. Value Health [Internet]. 2015 Jun 1 [cited 2024 Jun 12];18(4):512–29. Available from: https://pubmed.ncbi.nlm.nih.gov/26091606/. 10.1016/j.jval.2014.12.014 26091606

[43] Sculpher M, Fenwick E, Claxton K. Assessing quality in decision analytic cost-effectiveness models. A suggested framework and example of application. Pharmacoeconomics [Internet]. 2000 [cited 2024 Jun 12];17(5):461–77. Available from: https://pubmed.ncbi.nlm.nih.gov/10977388/. 10.2165/00019053-200017050-00005 10977388

[44] Soto J. Health economic evaluations using decision analytic modeling: Principles and Practices—Utilization of a checklist to their development and appraisal. Int J Technol Assess Health Care [Internet]. 2002 [cited 2024 Jun 12];18(1):94–111. Available from: https://www.cambridge.org/core/journals/international-journal-of-technology-assessment-in-health-care/article/health-economic-evaluations-using-decision-analytic-modeling/6F5FEF90246DEA875B77273FD7E47269. 11987445

[45] Adarkwah CC, van Gils PF, Hiligsmann M, Evers SMAA. Risk of bias in model-based economic evaluations: the ECOBIAS checklist. Expert Rev Pharmacoecon Outcomes Res [Internet]. 2016 Jul 3 [cited 2024 Jun 12];16(4):513–23. Available from: https://www.tandfonline.com/doi/abs/10.1586/14737167.2015.1103185. 10.1586/14737167.2015.1103185 26588001

[46] Dahabreh IJ, Trikalinos TA, Balk EM, Wong JB. Recommendations for the conduct and reporting of modeling and simulation studies in health technology assessment. Ann Intern Med [Internet]. 2016 Oct 18 [cited 2024 Jun 12];165(8):575–81. Available from: https://pubmed.ncbi.nlm.nih.gov/27750326/. 10.7326/M16-0161 27750326

[47] Catalá-López F, Ridao M, Alonso-Arroyo A, García-Altés A, Cameron C, González-Bermejo D, et al. The quality of reporting methods and results of cost-effectiveness analyses in Spain: a methodological systematic review. Syst Rev [Internet]. 2016 Jan 7 [cited 2024 Jun 12];5(1):1–11. Available from: 10.1186/s13643-015-0181-5. https://systematicreviewsjournal.biomedcentral.com/articles/. 10.1186/s13643-015-0181-5 PMC4731991 26822374

[48] Chaikledkaew U, Teerawattananon Y. Presentation of economic evaluation results. J Med Assoc Thai [Internet]. 2008;91 Suppl 2:S66-73. Available from: http://www.scopus.com/inward/record.url?scp=62449107331&partnerID=8YFLogxK. 19253489

[49] Abellán Perpiñán JM, Sánchez Martínez FI, Martínez Pérez JE. La medición de La Calidad de Los estudios de evaluación económica: Una propuesta de checklist Para La Toma de decisiones. Rev Esp Salud Publica. 2009;83:71–84. 19495490 10.1590/s1135-57272009000100006

[50] Akehurst R, Anderson P, Brazier J, Brennan A, Briggs A, Buxton M, et al. Decision analytic modelling in the economic evaluation of health technologies. Pharmacoeconomics [Internet]. 2000 Sep 21 [cited 2024 Jun 11];17(5):443–4. Available from: 10.2165/00019053-200017050-00003. https://link.springer.com/article/. 10.2165/00019053-200017050-00003 10977386

[51] Breeze PR, Squires H, Ennis K, Meier P, Hayes K, Lomax N, et al. Guidance on the use of complex systems models for economic evaluations of public health interventions. Health Econ [Internet]. 2023 Jul 1 [cited 2024 Jun 12];32(7):1603–25. Available from: https://pubmed.ncbi.nlm.nih.gov/37081811/. 10.1002/hec.4681 PMC10947434 37081811

[52] Drummond MF, Jefferson TO. Guidelines for authors and peer reviewers of economic submissions to the BMJ. The BMJ Economic Evaluation Working Party. BMJ [Internet]. 1996 [cited 2024 Jun 12];313(7052):275–83. Available from: https://pubmed.ncbi.nlm.nih.gov/8704542/. 10.1136/bmj.313.7052.275 PMC2351717 8704542

[53] Evers S, Goossens E, Andr´ A, Ament A, Banta D, Buxton M, et al. Criteria list for assessment of methodological quality of economic evaluations: consensus on health economic criteria. Int J Technol Assess Health Care [Internet]. 2005 [cited 2024 Jun 12];21(2):240–5. Available from: http://www.beoz.unimaas.nl/chec/. 15921065

[54] Schulenburg GVD, Greiner JM, Jost W, Klusen F, Kubin N, Leidl M. R, German recommendations on health economic evaluation: third and updated version of the Hanover Consensus. Value Health [Internet]. 2008 [cited 2024 Jun 12];11(4):539–44. Available from: https://pubmed.ncbi.nlm.nih.gov/18194408/. 10.1111/j.1524-4733.2007.00301.x 18194408

[55] Haji Ali Afzali H, Gray J, Karnon J. Model performance evaluation (validation and calibration) in model-based studies of therapeutic interventions for cardiovascular diseases: a review and suggested reporting framework. Appl Health Econ Health Policy [Internet]. 2013 Apr 28 [cited 2024 Jun 12];11(2):85–93. Available from: 10.1007/s40258-013-0012-6. https://link.springer.com/. 10.1007/s40258-013-0012-6 23456647

[56] Kunst N, Burger EA, Coupé VMH, Kuntz KM, Aas E. A Guide to an iterative approach to model-based decision making in health and medicine: an iterative decision-making framework. Pharmacoeconomics [Internet]. 2024 Apr 1 [cited 2024 Jun 12];42(4):363–71. Available from: https://pubmed.ncbi.nlm.nih.gov/38157129/. 10.1007/s40273-023-01341-z 38157129

[57] López-Bastida J, Oliva J, Antoñanzas F, García-Altés A, Gisbert R, Mar J, et al. Spanish recommendations on economic evaluation of health technologies. Eur J Health Econ. 2010;11(5):513–20. 20405159 10.1007/s10198-010-0244-4

[58] Marshall DA, Burgos-Liz L, Ijzerman MJ, Crown W, Padula WV, Wong PK, et al. Selecting a dynamic simulation modeling method for health care delivery research-part 2: report of the ISPOR dynamic simulation modeling emerging good practices task force. Value Health [Internet]. 2015 Mar 1 [cited 2024 Jun 12];18(2):147–60. Available from: https://pubmed.ncbi.nlm.nih.gov/25773550/. 10.1016/j.jval.2015.01.006 25773550

[59] Mauskopf J, Standaert B, Connolly MP, Culyer AJ, Garrison LP, Hutubessy R, et al. Economic analysis of vaccination programs: an ISPOR good practices for outcomes research task force report. Value Health [Internet]. 2018 Oct 1 [cited 2024 Jun 12];21(10):1133–49. Available from: https://pubmed.ncbi.nlm.nih.gov/30314613/. 10.1016/j.jval.2018.08.005 30314613

[60] McCabe C, Dixon S. Testing the validity of cost-effectiveness models. Pharmacoeconomics [Internet]. 2000 Sep 21 [cited 2024 Jun 12];17(5):501–13. Available from: https://link.springer.com/article/10.2165/00019053-200017050-00007. 10.2165/00019053-200017050-00007 10977390

[61] Petrou S, Gray A. Economic evaluation using decision analytical modelling: design, conduct, analysis, and reporting. BMJ [Internet]. 2011 May 28 [cited 2024 Jun 12];342(7808). Available from: https://pubmed.ncbi.nlm.nih.gov/21482590/. 10.1136/bmj.d1766 21482590

[62] Pokutnaya D, Childers B, Arcury-Quandt AE, Hochheiser H, Van Panhuis WG. An implementation framework to improve the transparency and reproducibility of computational models of infectious diseases. PLoS Comput Biol [Internet]. 2023 Mar 1 [cited 2024 Jun 12];19(3):e1010856. Available from: https://journals.plos.org/ploscompbiol/article?id=10.1371/journal.pcbi.1010856. 10.1371/journal.pcbi.1010856 PMC10019712 36928042

[63] Rosello A, Horner C, Hopkins S, Hayward AC, Deeny SR. Understanding the impact of interventions to prevent antimicrobial resistant infections in the long-term care facility: a review and practical guide to mathematical modeling. Infect Control Hosp Epidemiol [Internet]. 2017 Feb 1 [cited 2024 Jun 12];38(2):216–25. Available from: https://www.cambridge.org/core/journals/infection-control-and-hospital-epidemiology/article/understanding-the-impact-of-interventions-to-prevent-antimicrobial-resistant-infections-in-the-longterm-care-facility-a-review-and-practical-guide-to-mathematical-modeling/5B9252D248B672CB658FF21F637226D2. 10.1017/ice.2016.286 27989239

[64] Sanders GD, Neumann PJ, Basu A, Brock DW, Feeny D, Krahn M, et al. Recommendations for conduct, methodological practices, and reporting of cost-effectiveness analyses: second panel on cost-effectiveness in health and medicine. JAMA [Internet]. 2016 Sep 13 [cited 2024 Jun 12];316(10):1093–103. Available from: https://jamanetwork.com/journals/jama/fullarticle/2552214. 10.1001/jama.2016.12195 27623463

[65] Siegel JE, Weinstein MC, Russell LB, Gold MR. Recommendations for reporting cost-effectiveness analyses. Panel on cost-effectiveness in health and medicine. JAMA [Internet]. 1996 Oct 23 [cited 2024 Jun 12];276(16):1339–41. Available from: https://pubmed.ncbi.nlm.nih.gov/8861994/. 10.1001/jama.276.16.1339 8861994

[66] Silva E, Galvao T, Pereira M, Silva M. Estudos de avaliação econômica de tecnologias Em saúde: Roteiro Para análise crítica. Revista Panam De Salud Pública. 2014;35:219–27. 24793870

[67] Slayton RB, Program for the C for DC and, PMiH, O’Hagan JJ. Program for the C for DC and PMiH, Barnes S, Program for the C for DC and PMiH, Modeling Infectious Diseases in Healthcare Network (MInD-Healthcare) framework for describing and reporting multidrug-resistant organism and healthcare-associated infections agent-based modeling methods. Clin Infect Dis [Internet]. 2020 Dec 3 [cited 2024 Jun 12];71(9):2527–32. Available from: 10.1093/cid/ciaa234. 10.1093/cid/ciaa234 PMC7871347 32155235

[68] Thompson J, McClure R, Scott N, Hellard M, Abeysuriya R, Vidanaarachchi R, et al. A framework for considering the utility of models when facing tough decisions in public health: a guideline for policy-makers. Health Res Policy Syst [Internet]. 2022 Dec 1 [cited 2024 Jun 12];20(1):1–7. Available from: 10.1186/s12961-022-00902-6, https://health-policy-systems.biomedcentral.com/articles/. 10.1186/s12961-022-00902-6 PMC9547676 36209122

[69] Ultsch B, Damm O, Beutels P, Bilcke J, Brüggenjürgen B, Gerber-Grote A, et al. Methods for health economic evaluation of vaccines and immunization decision frameworks: a consensus framework from a European vaccine economics community. Pharmacoeconomics [Internet]. 2016 Mar 1 [cited 2024 Jun 12];34(3):227–44. Available from: https://pubmed.ncbi.nlm.nih.gov/26477039/. 10.1007/s40273-015-0335-2 PMC4766233 26477039

[70] Zawadzki RS, Gong CL, Cho SK, Schnitzer JE, Zawadzki NK, Hay JW, et al. Where do we go from here? A framework for using susceptible-infectious-recovered models for policy making in emerging infectious diseases. Value Health [Internet]. 2021 Jul 1 [cited 2024 Jun 12];24(7):917–24. Available from: https://pubmed.ncbi.nlm.nih.gov/34243834/. 10.1016/j.jval.2021.03.005 PMC8110035 34243834

[71] Langer A, Holle R, John J. Specific guidelines for assessing and improving the methodological quality of economic evaluations of newborn screening. BMC Health Serv Res [Internet]. 2012 [cited 2024 Jun 12];12(1). Available from: https://pubmed.ncbi.nlm.nih.gov/22947299/. 10.1186/1472-6963-12-300 PMC3459803 22947299

[72] Walter E, Zehetmayr S. Guidelines for health-economic evaluations in Austria. Wien Med Wochenschr [Internet]. 2006 Dec [cited 2024 Jun 12];156(23–24):628–32. Available from: https://pubmed.ncbi.nlm.nih.gov/17211768/. 10.1007/s10354-006-0360-z 17211768

[73] Heintz E, Gerber-Grote A, Ghabri S, Hamers FF, Rupel VP, Slabe-Erker R, et al. Is There a European view on health economic evaluations? Results from a synopsis of methodological guidelines used in the EUnetHTA partner countries. Pharmacoeconomics [Internet]. 2016 Jan 1 [cited 2024 Jun 12];34(1):59–76. Available from: 10.1007/s40273-015-0328-1. https://link.springer.com/. 10.1007/s40273-015-0328-1 26446858

