# Supplementary material for: Guidelines on reporting and assessing dynamic mathematical models of infectious diseases: a scoping review
Source: BMC Infect Dis. 2025 Dec 31;26:182. doi: 10.1186/s12879-025-12211-8 (PMC12849597; doi:10.1186/s12879-025-12211-8)
Supplement: Supplementary file 1 — Supplementary Material 1: Additional File 1: “Search strategy and additional results”. This file contains details on the search strategy as well as an analysis of results stratified by field (health economics, infectious diseases, and other). [file 12879_2025_12211_MOESM1_ESM.pdf]

Guidelines on reporting and assessing dynamic mathematical models of infectious diseases: A scoping review

Additional File 1. Search strategy and additional results

Contents

1.1. Literature search of guidelines and recommendations 1

1.1.1. Search results 1

1.1.2. Search strategies 1

1.1.2.1 MEDLINE (via OVID) 2

1.1.2.2. Web of Science 2

1.1.2.3 MedRxiv 3

1.1.3. Inclusion/Exclusion Criteria 4

1.2. Literature search for recent modelling studies 4

1.2.1 Search methodology and justification 4

1.2.2. Search results 5

1.2.3. Search strategy 5

1.2.4. Reference list 5

1.3. Additional Results 15

1.3.1. Modelling Field 15

1.3.2. Overview of dimensions and subdimensions 17

1.3.3. Prevalence of dimensions and subdimensions stratified by guideline 24

1.1. Literature search of guidelines and recommendations

1.1.1. Search results

**Date of search for all databases: 04.01.2024**	
**Database/Register**	**Hits**
**MEDLINE ALL (via Ovid)**<br>1946 to present	**3020**
**Web of Science (SCI-expanded, ESCI)**	**4516**
**MedRxiv BioRxiv**	**646**
**Total**	**8182**
**Total (after deduplication)**	**6319**

1.1.2. Search strategies

1.1.2.1 MEDLINE (via OVID)

The search timeframe included 1946 to January 03, 2024.

#	Searches
1	exp Guidelines as topic/ or exp Guideline/
2	(guideline* or guidance or (practice adj2 (guide*1 or recommend* or standard*)) or ((good* or best*) and practi*)).ti.
3	(framework* or checklist* or recommend* or concept* or standard* or position paper or reporting or reported or report?).ti.
4	or/1-3
5	((infecti* disease* or communicable disease* or transmission*) adj4 model*).tw.
6	(dynamic adj4 model*).ti.
7	(((economic* or cost?) adj2 evaluation*) or (cost? adj2 effectiveness)).ti.
8	((decision? adj1 analy*) and model*).tw.
9	(model* adj8 stud*).ti.
10	or/5-9
11	4 and 10

1.1.2.2. Web of Science

Science Citation Expanded 1945 to 2024, Emerging Sources Citation Index 2019 to 2024

#1	(((TI=(guideline*)) OR TI=(guidance*)) OR TI=((practice NEAR/2 (guide* or recommend* or standard*)))) OR TI=(((good* or best*) and practi*))
#2	TI=((framework* or checklist* OR recommend* OR concept* OR standard* OR "position paper" OR reporting OR reported OR report OR reports))
#3	#2 OR #1
#4	(TI=(("infecti* disease*" NEAR/4 model*) OR (communicable disease NEAR/4 model*) OR (transmission NEAR/4 model*))) OR AB=(("infecti* disease*" NEAR/4 model*) OR (communicable disease NEAR/4 model*) OR (transmission NEAR/4 model*))
#5	TI=((dynamic NEAR/4 model*))
#6	TI=(((economic* OR cost OR costs) NEAR/2 evaluation*) OR (cost NEAR/2 effectiveness) OR (costs NEAR/2 effectiveness))
#7	(TI=(((decision? NEAR/1 analy*) AND model*))) OR AB=(((decision? NEAR/1 analy*) AND model*))
#8	TI=((model* NEAR/8 stud*))

#9 #8 OR #7 OR #6 OR #5 OR #4

#10 #9 AND #3

1.1.2.3 MedRxiv

Advanced search in medRxiv or bioRxiv (https://www.medrxiv.org/search)

Separate searches in title or abstract

"infectious diseases" model* guideline*"; "communicable disease" model* guideline*"; "communicable diseases" model* guideline*"; "transmission* model* guideline*"; decision* model* guideline*; "economic evaluation" guideline*; "economic evaluations" guideline*; "cost evaluation guideline*"; "cost evaluations" guideline*"; "simulation model* guideline*"; "multi modal" comparison* guideline*"; "multimodal" comparison* guideline*"; "infectious diseases" framework* model*"; "communicable disease" model* framework*; "communicable diseases" model* framework*;

Separate searches in title:

"modelling guideline*"; "modeling guideline*"; "modelling guidance*"; "modeling guidance*";modeling* framework*; modelling* framework*; modeling* checklist*; modelling* checklist*; modelling* recommend*; modeling* recommend*; modelling* concept*; modeling* concept*; modelling "position paper"; modeling"position paper"; modelling report*; modelling report*; "infectious disease" model* guidance*; "infectious diseases" model* guidance*; "communicable disease" model* guidance*; "communicable diseases" model* guidance*; transmission* model* guidance*; decision* model* guidance*; "economic evaluation" guidance*; "economic evaluations" guidance*; cost evaluation guidance*; "cost evaluations" guidance*; simulation model* guidance*; "multi modal" comparison* guidance*; "multimodal" comparison* guidance*; "infectious disease" framework*; "transmission* framework*"; "decision* analytic* framework*"; ""economic evaluation" framework*"; ""economic evaluations" framework*"; ""cost evaluation" framework*"; ""cost evaluations" framework*"; ""multi modal" comparison* framework*"; ""multimodal" comparison* framework*"; ""infectious disease" model* checklist*"; ""infectious disease" checklist*"; ""communicable disease" checklist*"; ""communicable diseases" checklist*"; "transmission checklist*"; "decision model* checklist*"; "decision analytic* checklist*"; ""economic evaluation" checklist*"; ""economic evaluations" checklist*"; ""cost evaluation" checklist*"; ""cost evaluations" checklist*"; ""simulation model" checklist*"; ""multi modal comparison" checklist*"; ""multimodal comparison" checklist*"; ""infectious disease" recommend*"; ""infectious diseases" recommend*"; ""communicable disease" recommend*" ; ""communicable diseases" recommend*" ; "transmission model* recommend*"; "decision* model* recommend*"; "economic evaluation" recommend*; "economic evaluations" recommend*; ""cost evaluation" recommend*"; ""cost evaluations" recommend*"; "simulation model* recommend*"; ""multi modal comparison" recommend*"; ""multimodal comparison" recommend*"; ""infectious disease" concept*"; ""infectious diseases" concept*"; ""communicable disease" concept*"; ""communicable diseases" concept*"; "transmission model* concept*"; decision model* concept*; ""economic evaluation" concept*"; ""economic evaluations" concept*"; ""cost evaluation" concept*"; ""cost evaluations" concept*"; "multi modal comparison* concept*"; "multimodal comparison* concept*"; "infectious disease* standard*"; "communicable disease* standard*"; "transmission model* standard*"; "decision model* standard*"; "economic evaluation* standard*"; "simulation model* standard*"; "multi modal comparison* standard*";"multimodal comparison* standard*"; "position paper"; "disease* report*"; "transmission model* report*"; "decision model*

report*"; "economic evaluation* report*"; "cost* evaluation* report*"; "simulation model* report*"; "multi modal comparison* report*"; "multimodal comparison* report*"; "infectious disease* practi*"; "communicable disease* practi*"; "transmission* model* practi*"; "decision* model* practi*"; "economic evaluation* practi*"; "cost evaluation* practi*"; "simulation model* practi*"; ""multimodal" comparison* practi*"; ""multi-modal" comparison* practi*"

1.1.3. Inclusion/Exclusion Criteria

Inclusion	Exclusion
- Reporting and best practice guidelines for dynamic models or decision-analytic models<br>- Quality assessment tools for dynamic models or decision-analytic models	- Guidelines for clinical course models and drug or treatment assessments<br>- Non-generalisable or irrelevant (to infectious disease or decision-analytic model) guidelines<br>- Literature reviews unless they make suggestions of their own<br>- Errata to, replies to, comments on, or summaries of existing articles<br>- Conference abstracts<br>- Individual cost-effectiveness or modelling studies<br>- Articles presenting guidelines or recommendations which have subsequently been updated, for which the updated articles have been included<br>- Elaboration and Explanation articles accompanying included articles<br>- Non-English articles for which the English version has been included<br>- Articles that cover only one single dimension

1.2. Literature search for recent modelling studies

1.2.1 Search methodology and justification

In addition to our main literature search (explained above in section 1.1. of Additional File 1), we wanted to gain some insight into whether infectious disease modelling studies report using reporting guidelines. To get an initial impression of this, we searched MEDLINE ALL (via Ovid) for infectious disease modelling studies published between 1st January 2019 and 22nd January 2024 (details of the search strategy can be found in section 1.2.3. of Additional File 1). A random selection (obtained via a random number generator) of 500 of the 9854 hits then underwent single-screening (MC) of the titles and abstracts to identify articles which actually covered infectious disease modelling studies. This screening process resulted in a list of 100 publications (see section 1.2.4 of Additional File 1). We then went through these 100 recent modelling publications to establish if any of the authors mentioned following a reporting guideline (AB, VKJ). Any guidelines identified via this strategy which were deemed relevant and followed the inclusion/exclusion criteria of our main search (see section 1.1.3. of Additional File 1) were included in the list of articles to be extracted.

Only one modelling study (1) of the 100 we searched through mentioned using a reporting guideline (2). This reporting guideline has subsequently been updated, so the updated version (3) was

included in the scoping review and was thus extracted, as per the procedure used for updated guidelines identified via the main search.

1. Wallentin G, Kaziyeva D, Reibersdorfer-Adelsberger E. COVID-19 Intervention Scenarios for a Long-term Disease Management. Int J Health Policy Manag [Internet]. 2020 Dec 1 [cited 2024 May 29];9(12):508. Available from: https://doi.org/10.34172/ijhpm.2020.130

2. Grimm V, Berger U, DeAngelis DL, Polhill JG, Giske J, Railsback SF. The ODD protocol: A review and first update. Ecol Modell. 2010 Nov 24;221(23):2760–8.

3. Grimm V, Railsback SF, Vincenot CE, Berger U, Gallagher C, DeAngelis DL, et al. The ODD Protocol for Describing Agent-Based and Other Simulation Models: A Second Update to Improve Clarity, Replication, and Structural Realism. Journal of Artificial Societies and Social Simulation [Internet]. 2020 [cited 2024 Dec 4];23(2). Available from: http://jasss.soc.surrey.ac.uk/23/2/7.html

1.2.2. Search results

**Date of search for all databases: 22.01.2024**	
**Database/Register**	**Search**
**MEDLINE ALL (via Ovid)**<br>1946 to present	**9898**
**Total (after deduplication)**	**9854**

1.2.3. Search strategy

A single search was conducted on MEDLINE ALL (via Ovid) covering the time period from 01.01.2019 to 22.01.2024.

1 Communicable Diseases/ep, pc, tm [Epidemiology, Prevention & Control, Transmission]

2 Disease Transmission, Infectious/pc [Prevention & Control]

3 (pandemic* or infectious disease* or epidemic* or communicable disease*).tw.

4 or/1-3

5 (peak? or (scenario? adj1 (future or projection*)) or outbreak or transmission?).tw.

6 (model or modelling or modeling or forecast or forecasts or forecasting or random forest* or nowcasting*).tw.

7 4 and 5and 6

8 limit 7 to yr="2019-Current"

1.2.4. Reference list

The 100 studies which were included are listed here:

1. Yuan H, Kramer SC, Lau EHY, Cowling BJ, Yang W. Modeling influenza seasonality in the tropics and subtropics. PLOS Computational Biology [Internet]. 2021 Jun 1 [cited 2024 May 29];17(6):e1009050. Available from: https://journals.plos.org/ploscompbiol/article?id=10.1371/journal.pcbi.1009050

2. Zang X, Krebs E, Mah C, Min JE, Marshall BDL, Feaster DJ, et al. Can the "Ending the HIV Epidemic" initiative transition the USA towards HIV/AIDS epidemic control? AIDS (London, England) [Internet]. 2020 Dec 1 [cited 2024 May 29];34(15):2325–8. Available from: https://pubmed.ncbi.nlm.nih.gov/32796216/

3. Zhong H, Wang W. Mathematical modelling for scarlet fever with direct and indirect infections. Journal of biological dynamics [Internet]. 2020 Jan 1 [cited 2024 May 29];14(1):767–87. Available from: https://pubmed.ncbi.nlm.nih.gov/33063646/

4. Zhou W, Xiao Y, Heffernan JM. Optimal media reporting intensity on mitigating spread of an emerging infectious disease. PLoS ONE [Internet]. 2019 Mar 1 [cited 2024 May 29];14(3). Available from: /pmc/articles/PMC6428274/

5. Yang Z, Zeng Z, Wang K, Wong SS, Liang W, Zanin M, et al. Modified SEIR and AI prediction of the epidemics trend of COVID-19 in China under public health interventions. Journal of thoracic disease [Internet]. 2020 Mar 1 [cited 2024 May 29];12(3):165–74. Available from: https://pubmed.ncbi.nlm.nih.gov/32274081/

6. Yang S, Guo X, Zhao Z, Abudunaibi B, Zhao Y, Rui J, et al. Possibility of mpox viral transmission and control from high-risk to the general population: a modeling study. BMC infectious diseases [Internet]. 2023 Dec 1 [cited 2024 May 29];23(1). Available from: https://pubmed.ncbi.nlm.nih.gov/36829116/

7. Xu C, Cheng K, Wang Y, Liu M, Wang X, Yang Z, et al. Analysis of the current status of TB transmission in China based on an age heterogeneity model. Mathematical biosciences and engineering : MBE [Internet]. 2023 [cited 2024 May 29];20(11):19232–523. Available from: https://pubmed.ncbi.nlm.nih.gov/38052598/

8. Xiridou M, Heijne J, Adam P, de Coul EO, Matser A, de Wit J, et al. How the Disruption in Sexually Transmitted Infection Care Due to the COVID-19 Pandemic Could Lead to Increased Sexually Transmitted Infection Transmission Among Men Who Have Sex With Men in The Netherlands: A Mathematical Modeling Study. Sexually transmitted diseases [Internet]. 2022 Feb 1 [cited 2024 May 29];49(2):145–53. Available from: https://pubmed.ncbi.nlm.nih.gov/34475357/

9. Wilk AM, Łakomiec K, Psiuk-Maksymowicz K, Fujarewicz K. Impact of government policies on the COVID-19 pandemic unraveled by mathematical modelling. Scientific Reports 2022 12:1 [Internet]. 2022 Oct 10 [cited 2024 May 29];12(1):1–13. Available from: https://www.nature.com/articles/s41598-022-21126-2

10. Wang X, Wang S, Wang J, Rong L. A Multiscale Model of COVID-19 Dynamics. Bulletin of Mathematical Biology [Internet]. 2022 Sep 1 [cited 2024 May 29];84(9):99. Available from: /pmc/articles/PMC9360740/

11. Wang L, Wang J, Zhao H, Shi Y, Wang K, Wu P, et al. Modelling and assessing the effects of medical resources on transmission of novel coronavirus (COVID-19) in Wuhan, China. Mathematical biosciences and engineering : MBE [Internet]. 2020 Mar 30 [cited 2024 May 29];17(4):2936–49. Available from: https://pubmed.ncbi.nlm.nih.gov/32987508/

12. Wang ST, Li L, Zhang J, Li Y, Luo XF, Sun GQ. Quantitative evaluation of the role of Fangcang shelter hospitals in the control of Omicron transmission: A case study of the outbreak in Shanghai, China in 2022. One Health. 2023 Jun 1;16:100475.

13. Wallentin G, Kaziyeva D, Reibersdorfer-Adelsberger E. COVID-19 Intervention Scenarios for a Long-term Disease Management. International Journal of Health Policy and Management [Internet]. 2020 Dec 1 [cited 2024 May 29];9(12):508. Available from: /pmc/articles/PMC7947653/

14. van Wyk H, Eisenberg JNS, Brouwer AF. Long-term projections of the impacts of warming temperatures on Zika and dengue risk in four Brazilian cities using a temperature-dependent basic reproduction number. PLoS neglected tropical diseases [Internet]. 2023 Apr 1 [cited 2024 May 29];17(4). Available from: https://pubmed.ncbi.nlm.nih.gov/37104296/

15. Valiati NCM, Villela DAM. Modelling policy combinations of vaccination and transmission suppression of SARS-CoV-2 in Rio de Janeiro, Brazil. Infectious Disease Modelling [Internet]. 2022 Mar 1 [cited 2024 May 29];7(1):231–42. Available from: https://pubmed.ncbi.nlm.nih.gov/35005325/

16. Tang B, Xia F, Tang S, Bragazzi NL, Li Q, Sun X, et al. The effectiveness of quarantine and isolation determine the trend of the COVID-19 epidemics in the final phase of the current outbreak in China. International Journal of Infectious Diseases. 2020 Jun 1;95:288–93.

17. Tang B, Xia F, Bragazzi NL, McCarthy Z, Wang X, He S, et al. Lessons drawn from China and South Korea for managing COVID-19 epidemic: Insights from a comparative modeling study. ISA Transactions. 2022 May 1;124:164–75.

18. Tadić B, Melnik R. Microscopic dynamics modeling unravels the role of asymptomatic virus carriers in SARS-CoV-2 epidemics at the interplay between biological and social factors. Computers in Biology and Medicine [Internet]. 2021 Jun 1 [cited 2024 May 29];133:104422. Available from: /pmc/articles/PMC8078086/

19. Šušteršič T, Blagojević A, Cvetković D, Cvetković A, Lorencin I, Šegota SB, et al. Epidemiological Predictive Modeling of COVID-19 Infection: Development, Testing, and Implementation on the Population of the Benelux Union. Frontiers in public health [Internet]. 2021 Oct 28 [cited 2024 May 29];9. Available from: https://pubmed.ncbi.nlm.nih.gov/34778171/

20. Sun D, Duan L, Xiong J, Wang D. Modeling and forecasting the spread tendency of the COVID-19 in China. Advances in Difference Equations [Internet]. 2020 Dec 1 [cited 2024 May 29];2020(1):489. Available from: /pmc/articles/PMC7487449/

21. Spannaus A, Papamarkou T, Erwin S, Christian JB. Inferring the spread of COVID-19: the role of time-varying reporting rate in epidemiological modelling. Scientific Reports 2022 12:1 [Internet]. 2022 Jun 24 [cited 2024 May 29];12(1):1–12. Available from: https://www.nature.com/articles/s41598-022-14979-0

22. Siraj A, Worku A, Berhane K, Aregawi M, Eshetu M, Mirkuzie A, et al. Early estimates of COVID-19 infections in small, medium and large population clusters. BMJ Global Health [Internet]. 2020 Sep 18 [cited 2024 May 29];5(9):3055. Available from: /pmc/articles/PMC7503195/

23. Shen M, Zu J, Fairley CK, Pagán JA, Ferket B, Liu B, et al. Effects of New York's Executive Order on Face Mask Use on COVID-19 Infections and Mortality: A Modeling Study. Journal of Urban Health : Bulletin of the New York Academy of Medicine [Internet]. 2021 Apr 1 [cited 2024 May 29];98(2):197. Available from: /pmc/articles/PMC7919630/

24. Saunders HA, Schwartz JM. COVID-19 vaccination strategies depend on the underlying network of social interactions. Scientific Reports 2021 11:1 [Internet]. 2021 Dec 15 [cited 2024 May 29];11(1):1–10. Available from: https://www.nature.com/articles/s41598-021-03167-1

25. Salvatore PP, Kendall EA, Seabrook D, Brown J, Durham GH, Dowdy DW. Projecting the impact of variable MDR-TB transmission efficiency on long-term epidemic trends in South Africa and Vietnam. Scientific Reports 2019 9:1 [Internet]. 2019 Dec 2 [cited 2024 May 29];9(1):1–12. Available from: https://www.nature.com/articles/s41598-019-54561-9

26. Salvador R, Tanquilut N, Macmac R, Lampang KN, Chaisowwong W, Pfeiffer D, et al. Evaluation of strategies using simulation model to control a potential outbreak of highly pathogenic avian influenza among poultry farms in Central Luzon, Philippines. PLOS ONE [Internet]. 2020 Sep 1 [cited 2024 May 29];15(9):e0238815. Available from: https://journals.plos.org/plosone/article?id=10.1371/journal.pone.0238815

27. Rozhnova G, van Dorp CH, Bruijning-Verhagen P, Bootsma MCJ, van de Wijgert JHHM, Bonten MJM, et al. Model-based evaluation of school- and non-school-related measures to control the COVID-19 pandemic. Nature Communications 2021 12:1 [Internet]. 2021 Mar 12 [cited 2024 May 29];12(1):1–11. Available from: https://www.nature.com/articles/s41467-021-21899-6

28. Saikia D, Bora K, Bora MP. COVID-19 outbreak in India: an SEIR model-based analysis. Nonlinear dynamics [Internet]. 2021 Jun 1 [cited 2024 May 29];104(4):4727–51. Available from: https://pubmed.ncbi.nlm.nih.gov/34108815/

29. Roosa K, Tariq A, Yan P, Hyman JM, Chowell G. Multi-model forecasts of the ongoing Ebola epidemic in the Democratic Republic of Congo, March–October 2019. Journal of the Royal Society Interface [Internet]. 2020 Aug 18 [cited 2024 May 29];17(169). Available from: https://royalsocietypublishing.org/doi/10.1098/rsif.2020.0447

30. Rizwan-Ul-Hasan S, Farrukh F, Ahmed S, Abidi SH. A mathematical modeling approach to measure the probability of HIV-1 transmission for different high-risk groups of Pakistan. Journal of infection in developing countries [Internet]. 2021 Aug 31 [cited 2024 May 29];15(8):1212–5. Available from: https://pubmed.ncbi.nlm.nih.gov/34516431/

31. Rehman AU, Mian SH, Usmani YS, Abidi MH, Mohammed MK. Modeling Consequences of COVID-19 and Assessing Its Epidemiological Parameters: A System Dynamics Approach. Healthcare (Basel, Switzerland) [Internet]. 2023 Jan 1 [cited 2024 May 29];11(2). Available from: https://pubmed.ncbi.nlm.nih.gov/36673628/

32. Reddy KP, Shebl FM, Foote JHA, Harling G, Scott JA, Panella C, et al. Cost-effectiveness of public health strategies for COVID-19 epidemic control in South Africa: a microsimulation modelling study. The Lancet Global health [Internet]. 2021 Feb 1 [cited 2024 May 29];9(2):e120–9. Available from: https://pubmed.ncbi.nlm.nih.gov/33188729/

33. Quiner C, Jones K, Bobashev G. Impacts of timing, length, and intensity of behavioral interventions to COVID-19 dynamics: North Carolina county-level examples. Infectious Disease Modelling. 2022 Sep 1;7(3):535–44.

34. Prasad N, Read JM, Jewell C, Waite B, Trenholme AA, Huang QS, et al. Modelling the impact of respiratory syncytial virus (RSV) vaccine and immunoprophylaxis strategies in New Zealand. Vaccine. 2021 Jul 13;39(31):4383–90.

35. Prada JM, Stolk WA, Davis EL, Touloupou P, Sharma S, Munoz J, et al. Delays in lymphatic filariasis elimination programmes due to COVID-19, and possible mitigation strategies. Transactions of the Royal Society of Tropical Medicine and Hygiene [Internet]. 2021 Mar 1 [cited 2024 May 29];115(3):261. Available from: /pmc/articles/PMC7928650/

36. Potluri R, Kumar A, Oriol-mathieu V, van Effelterre T, Metz L, Bhandari H. Model-based evaluation of the impact of prophylactic vaccination applied to Ebola epidemics in Sierra

Leone and Democratic Republic of Congo. BMC infectious diseases [Internet]. 2022 Dec 1 [cited 2024 May 29];22(1). Available from: https://pubmed.ncbi.nlm.nih.gov/36192683/

37. Pons-Salort M, John J, Watson OJ, Brazeau NF, Verity R, Kang G, et al. Reassessing Reported Deaths and Estimated Infection Attack Rate during the First 6 Months of the COVID-19 Epidemic, Delhi, India. Emerging Infectious Diseases [Internet]. 2022 Apr 1 [cited 2024 May 29];28(4):759. Available from: /pmc/articles/PMC8962916/

38. Paltiel AD, Zheng A, Walensky RP. COVID-19 screening strategies that permit the safe re-opening of college campuses. medRxiv [Internet]. 2020 Jul 7 [cited 2024 May 29];(203):436–4081. Available from: /pmc/articles/PMC7359539/

39. Nuraini N, Fauzi IS, Lestari BW, Rizqina S. The Impact of COVID-19 Quarantine on Tuberculosis and Diabetes Mellitus Cases: A Modelling Study. Tropical Medicine and Infectious Disease [Internet]. 2022 Dec 1 [cited 2024 May 29];7(12). Available from: /pmc/articles/PMC9782997/

40. Nielsen BF, Eilersen A, Simonsen L, Sneppen K. Lockdowns exert selection pressure on overdispersion of SARS-CoV-2 variants. Epidemics. 2022 Sep 1;40:100613.

41. Ngonghala CN, Ryan SJ, Tesla B, Demakovsky LR, Mordecai EA, Murdock CC, et al. Effects of changes in temperature on Zika dynamics and control. Journal of the Royal Society Interface [Internet]. 2021 May 5 [cited 2024 May 29];18(178). Available from: https://royalsocietypublishing.org/doi/10.1098/rsif.2021.0165

42. Ng TC, Cheng HY, Chang HH, Liu CC, Yang CC, Jian SW, et al. Comparison of Estimated Effectiveness of Case-Based and Population-Based Interventions on COVID-19 Containment in Taiwan. JAMA internal medicine [Internet]. 2021 Jul 1 [cited 2024 May 29];181(7):913–21. Available from: https://pubmed.ncbi.nlm.nih.gov/33821922/

43. Naffeti B, BenAribi W, Kebir A, Diarra M, Schoenhals M, Vigan-Womas I, et al. Comparative reconstruction of SARS-CoV-2 transmission in three African countries using a mathematical model integrating immunity data. IJID regions [Internet]. 2023 Mar 1 [cited 2024 May 29];10:100–7. Available from: https://pubmed.ncbi.nlm.nih.gov/38204927/

44. Morsky B, Magpantay F, Day T, Akçay E. The impact of threshold decision mechanisms of collective behavior on disease spread. Proceedings of the National Academy of Sciences of the United States of America [Internet]. 2023 May 9 [cited 2024 May 29];120(19):e2221479120. Available from: https://www.pnas.org/doi/abs/10.1073/pnas.2221479120

45. López JAM, García BA, Bentkowski P, Bioglio L, Pinotti F, Boëlle PY, et al. Anatomy of digital contact tracing: Role of age, transmission setting, adoption, and case detection. Science Advances [Internet]. 2021 Apr 9 [cited 2024 May 29];7(15). Available from: /pmc/articles/PMC8034853/

46. Moghadas SM, Vilches TN, Zhang K, Wells CR, Shoukat A, Singer BH, et al. The impact of vaccination on COVID-19 outbreaks in the United States. medRxiv [Internet]. 2020 Nov 30 [cited 2024 May 29]; Available from: /pmc/articles/PMC7709178/

47. Mettler SK, Park J, Özbek O, Mettler LK, Ho PH, Rhim HC, et al. The importance of timely contact tracing — A simulation study. International Journal of Infectious Diseases. 2021 Jul 1;108:309–19.

48. Malek A, Hoque A. Mathematical modeling of bird flu with vaccination and treatment for the poultry farms. Comparative immunology, microbiology and infectious diseases [Internet]. 2022 Jan 1 [cited 2024 May 29];80. Available from: https://pubmed.ncbi.nlm.nih.gov/34891070/

49. Lucia-Sanz A, Magalie A, Rodriguez-Gonzalez R, Leung CY, Weitz JS. Modeling shield immunity to reduce COVID-19 transmission in long-term care facilities. Annals of Epidemiology. 2023 Jan 1;77:44–52.

50. Luangasanatip N, Painter C, Pan-ngum W, Saralamba S, Wichaita T, White L, et al. How to model the impact of vaccines for policymaking when the characteristics are uncertain: A case study in Thailand prior to the vaccine rollout during the COVID-19 pandemic. Vaccine. 2023 Jul 25;41(33):4854–60.

51. Liu J, Cao L, Zhang D, Chen Z, Lian X, Li Y, et al. Optimization of Site Selection for Emergency Medical Facilities considering the SEIR Model. Computational intelligence and neuroscience [Internet]. 2022 [cited 2024 May 29];2022. Available from: https://pubmed.ncbi.nlm.nih.gov/35463288/

52. Liang S, Jiang T, Jiao Z, Zhou Z. A model simulation on the SARS-CoV-2 Omicron variant containment in Beijing, China. Intelligent Medicine [Internet]. 2023 Feb 1 [cited 2024 May 29];3(1):10. Available from: /pmc/articles/PMC9677562/

53. Li W, Gong J, Zhou J, Zhang L, Wang D, Li J, et al. An evaluation of COVID-19 transmission control in Wenzhou using a modified SEIR model. Epidemiology and Infection [Internet]. 2021 [cited 2024 May 29];149. Available from: /pmc/articles/PMC7804084/

54. Leung K, Wu JT, Leung GM. Effects of adjusting public health, travel, and social measures during the roll-out of COVID-19 vaccination: a modelling study. The Lancet Public Health [Internet]. 2021 Sep 1 [cited 2024 May 29];6(9):e674–82. Available from: http://www.thelancet.com/article/S2468266721001675/fulltext

55. Lee F, Khanna AS, Hallmark CJ, Lavingia R, McNeese M, Zhao J, et al. Expanding Medicaid to Reduce Human Immunodeficiency Virus Transmission in Houston, Texas: Insights From a Modeling Study. Medical Care [Internet]. 2023 Jan 1 [cited 2024 May 29];61(1):12. Available from: /pmc/articles/PMC9733589/

56. Koltai M, Krauer F, Hodgson D, van Leeuwen E, Treskova-Schwarzbach M, Jit M, et al. Determinants of RSV epidemiology following suppression through pandemic contact restrictions. Epidemics [Internet]. 2022 Sep 1 [cited 2024 May 29];40:100614. Available from: /pmc/articles/PMC9301974/

57. Kinoshita R, Anzai A, Jung SM, Linton NM, Miyama T, Kobayashi T, et al. Containment, Contact Tracing and Asymptomatic Transmission of Novel Coronavirus Disease (COVID-19): A Modelling Study. Journal of clinical medicine [Internet]. 2020 Oct 1 [cited 2024 May 29];9(10):1–9. Available from: https://pubmed.ncbi.nlm.nih.gov/32992614/

58. Kim HY, Bershteyn A, McGillen JB, Shaff J, Sisti J, Ko C, et al. Social distancing and mask-wearing could avoid recurrent stay-at-home restrictions during COVID-19 respiratory pandemic in New York City. Scientific Reports 2022 12:1 [Internet]. 2022 Jun 20 [cited 2024 May 29];12(1):1–10. Available from: https://www.nature.com/articles/s41598-022-13310-1

59. Khan T, Rihan FA, Ahmad H. Modelling the dynamics of acute and chronic hepatitis B with optimal control. Scientific Reports 2023 13:1 [Internet]. 2023 Sep 11 [cited 2024 May 29];13(1):1–15. Available from: https://www.nature.com/articles/s41598-023-39582-9

60. Jijón S, Molina JM, Costagliola D, Supervie V, Breban R. Can HIV epidemics among MSM be eliminated through participation in preexposure prophylaxis rollouts? AIDS (London, England) [Internet]. 2021 Nov 15 [cited 2024 May 29];35(14):2347–54. Available from: https://pubmed.ncbi.nlm.nih.gov/34224442/

61. Iboi EA, Sharomi O, Ngonghala CN, Gumel AB. Mathematical modeling and analysis of COVID-19 pandemic in Nigeria. Mathematical biosciences and engineering : MBE [Internet]. 2020 Oct 22 [cited 2024 May 29];17(6):7192–220. Available from: https://pubmed.ncbi.nlm.nih.gov/33378893/

62. Head JR, Andrejko KL, Cheng Q, Collender PA, Phillips S, Boser A, et al. School closures reduced social mixing of children during COVID-19 with implications for transmission risk and school reopening policies. Journal of the Royal Society Interface [Internet]. 2021 Apr 1 [cited 2024 May 29];18(177). Available from: https://royalsocietypublishing.org/doi/10.1098/rsif.2020.0970

63. Gustin MP, Pujo-Menjouet L, Vanhems P. Influenza transmissibility among patients and health-care professionals in a geriatric short-stay unit using individual contact data. Scientific reports [Internet]. 2023 Dec 1 [cited 2024 May 29];13(1). Available from: https://pubmed.ncbi.nlm.nih.gov/37386032/

64. Guo Y, Ye W, Zhao Z, Guo X, Song W, Su Y, et al. Simulating potential outbreaks of Delta and Omicron variants based on contact-tracing data: A modelling study in Fujian Province, China. Infectious Disease Modelling [Internet]. 2023 Mar 1 [cited 2024 May 29];8(1):270–81. Available from: https://pubmed.ncbi.nlm.nih.gov/36846047/

65. Guo K, Lu Y, Geng Y, Lu J, Shi L. Assessing the medical resources in COVID-19 based on evolutionary game. PLOS ONE [Internet]. 2023 Jan 1 [cited 2024 May 29];18(1). Available from: /pmc/articles/PMC9833555/

66. Gomez LM, Meszaros VA, Turner WC, Brandon Ogbunugafor C. The Epidemiological Signature of Pathogen Populations That Vary in the Relationship between Free-Living Parasite Survival and Virulence. Viruses [Internet]. 2020 Sep 1 [cited 2024 May 29];12(9). Available from: https://pubmed.ncbi.nlm.nih.gov/32971954/

67. George A, Li C, Lim JZ, Xie T. From SARS to COVID-19: The evolving role of China-ASEAN production network. Economic Modelling. 2021 Aug 1;101:105510.

68. Gavish N, Yaari R, Huppert A, Katriel G. Population-level implications of the Israeli booster campaign to curtail COVID-19 resurgence. Science translational medicine [Internet]. 2022 Jun 1 [cited 2024 May 29];14(647). Available from: https://pubmed.ncbi.nlm.nih.gov/35412326/

69. Razi A, Friston KJ, Parr T, Zeidman P, Flandin G, Daunizeau J, et al. Second waves, social distancing, and the spread of COVID-19 across the USA. Wellcome Open Research [Internet]. 2020 [cited 2024 May 29];5. Available from: /pmc/articles/PMC8063524/

70. Franco C, Ferreira LS, Sudbrack V, Borges ME, Poloni S, Prado PI, et al. Percolation across households in mechanistic models of non-pharmaceutical interventions in SARS-CoV-2 disease dynamics. Epidemics. 2022 Jun 1;39:100551.

71. Feng S, Feng Z, Ling C, Chang C, Feng Z. Prediction of the COVID-19 epidemic trends based on SEIR and AI models. PLoS ONE [Internet]. 2021 Jan 1 [cited 2024 May 29];16(1). Available from: /pmc/articles/PMC7793264/

72. Ertem Z, Araz OM, Cruz-Aponte M. A decision analytic approach for social distancing policies during early stages of COVID-19 pandemic. Decision Support Systems. 2022 Oct 1;161:113630.

73. Djiomba Njankou SD, Nyabadza F. Modelling the Role of Human Behaviour in Ebola Virus Disease (EVD) Transmission Dynamics. Computational and Mathematical Methods in Medicine [Internet]. 2022 [cited 2024 May 29];2022. Available from: /pmc/articles/PMC9122724/

74. Derjany P, Namilae S, Liu D, Srinivasan A. Multiscale model for the optimal design of pedestrian queues to mitigate infectious disease spread. PloS one [Internet]. 2020 Jul 1 [cited 2024 May 29];15(7). Available from: https://pubmed.ncbi.nlm.nih.gov/32645057/

75. Compagni RD, Cheng Z, Russo S, van Boeckel TP. A hybrid Neural Network-SEIR model for forecasting intensive care occupancy in Switzerland during COVID-19 epidemics. PLOS ONE [Internet]. 2022 Mar 1 [cited 2024 May 29];17(3):e0263789. Available from: https://journals.plos.org/plosone/article?id=10.1371/journal.pone.0263789

76. Delamater PL, Woodul RL. NC-COVID: A Time-Varying Compartmental Model for Estimating SARS-CoV-2 Infection Dynamics in North Carolina, US. medRxiv [Internet]. 2022 Oct 25 [cited 2024 May 29]; Available from: /pmc/articles/PMC9628207/

77. da Cruz PPA, Crema-Cruz LC, Campos FS. Modeling transmission dynamics of severe acute respiratory syndrome coronavirus 2 in São Paulo, Brazil. Revista da Sociedade Brasileira de Medicina Tropical [Internet]. 2021 [cited 2024 May 29];54:1–8. Available from: https://pubmed.ncbi.nlm.nih.gov/33533818/

78. Cousien A, Abel S, Monthieux A, Andronico A, Calmont I, Cervantes M, et al. Assessing Zika Virus Transmission Within Households During an Outbreak in Martinique, 2015-2016. American journal of epidemiology [Internet]. 2019 Jul 1 [cited 2024 May 29];188(7):1389–96. Available from: https://pubmed.ncbi.nlm.nih.gov/30995296/

79. Chung GS, Hutton DW. Epidemiological impact and cost-effectiveness of universal meningitis b vaccination among college students prior to college entry. PloS one [Internet]. 2020 Oct 1 [cited 2024 May 29];15(10). Available from: https://pubmed.ncbi.nlm.nih.gov/33035260/

80. Choi S, Ki M. Estimating the reproductive number and the outbreak size of COVID-19 in Korea. Epidemiology and Health [Internet]. 2020 [cited 2024 May 28];42. Available from: /pmc/articles/PMC7285447/

81. Childs ML, Kain MP, Harris MJ, Kirk D, Couper L, Nova N, et al. The impact of long-term non-pharmaceutical interventions on COVID-19 epidemic dynamics and control: the value and limitations of early models. Proceedings of the Royal Society B [Internet]. 2021 Aug 25 [cited 2024 May 28];288(1957). Available from: https://royalsocietypublishing.org/doi/10.1098/rspb.2021.0811

82. Chen B, Zhao Y, Jin Z, He D, Li H. Twice evasions of Omicron variants explain the temporal patterns in six Asian and Oceanic countries. BMC Infectious Diseases [Internet]. 2023 Dec 1 [cited 2024 May 28];23(1):1–12. Available from: https://bmcinfectdis.biomedcentral.com/articles/10.1186/s12879-023-07984-9

83. Cepeda JA, Vickerman P, Bruneau J, Zang G, Borquez A, Farrell M, et al. Estimating the contribution of stimulant injection to HIV and HCV epidemics among people who inject drugs and implications for harm reduction: A modeling analysis. Drug and Alcohol Dependence. 2020 Aug 1;213:108135.

84. Calvetti D, Somersalo E. Post-pandemic modeling of COVID-19: Waning immunity determines recurrence frequency. Mathematical biosciences [Internet]. 2023 Nov 1 [cited 2024 May 28];365. Available from: https://pubmed.ncbi.nlm.nih.gov/37708989/

85. Burns AAC, Gutfraind A. Effectiveness of Isolation Policies in Schools: Evidence from a Mathematical Model of Influenza and COVID-19. medRxiv [Internet]. 2020 Nov 23 [cited 2024 May 28]; Available from: /pmc/articles/PMC7276029/

86. Brugger J, Althaus CL. Transmission of and susceptibility to seasonal influenza in Switzerland from 2003 to 2015. Epidemics. 2020 Mar 1;30:100373.

87. Brainard J, Hunter PR. Misinformation making a disease outbreak worse: outcomes compared for influenza, monkeypox, and norovirus. Simulation [Internet]. 2020 Apr 1 [cited 2024 May 28];96(4):365. Available from: /pmc/articles/PMC8282656/

88. Becker DJ, Ketterson ED, Hall RJ. Reactivation of latent infections with migration shapes population-level disease dynamics. Proceedings of the Royal Society B [Internet]. 2020 Sep 1 [cited 2024 May 28];287(1935). Available from: https://royalsocietypublishing.org/doi/10.1098/rspb.2020.1829

89. Boyer CB, Rumpler E, Kissler SM, Lipsitch M. Infectious disease dynamics and restrictions on social gathering size. Epidemics [Internet]. 2022 Sep 1 [cited 2024 May 28];40:100620. Available from: /pmc/articles/PMC9384337/

90. Bentout S, Chekroun A, Kuniya T. Parameter estimation and prediction for coronavirus disease outbreak 2019 (COVID-19) in Algeria. AIMS Public Health [Internet]. 2020 [cited 2024 May 28];7(2):306. Available from: /pmc/articles/PMC7327392/

91. Balabdaoui F, Mohr D. Age-stratified discrete compartment model of the COVID-19 epidemic with application to Switzerland. Scientific Reports 2020 10:1 [Internet]. 2020 Dec 4 [cited 2024 May 28];10(1):1–12. Available from: https://www.nature.com/articles/s41598-020-77420-4

92. Bajiya VP, Bugalia S, Tripathi JP. Mathematical modeling of COVID-19: Impact of non-pharmaceutical interventions in India. Chaos (Woodbury, NY) [Internet]. 2020 Nov 1 [cited 2024 May 28];30(11). Available from: https://pubmed.ncbi.nlm.nih.gov/33261327/

93. Aziz MHN, Safaruddin ADA, Hamzah NA, Supadi SS, Yuhao Z, Aziz MA. Modelling the Effect of Vaccination Program and Inter-state Travel in the Spread of COVID-19 in Malaysia. Acta Biotheoretica [Internet]. 2023 Mar 1 [cited 2024 May 28];71(1):1–22. Available from: https://link.springer.com/article/10.1007/s10441-022-09453-3

94. Asamoah J, ... Sensitivity assessment and optimal economic evaluation of a new COVID-19 compartmental epidemic model with control interventions - ScienceDirect [Internet]. 2021 [cited 2024 May 28]. Available from:https://www.sciencedirect.com/science/article/pii/S0960077921002381

95. Ayoub HH, Chemaitelly H, Seedat S, Mumtaz GR, Makhoul M, Abu-Raddad LJ. Age could be driving variable SARS-CoV-2 epidemic trajectories worldwide. PLOS ONE [Internet]. 2020 Aug 1 [cited 2024 May 28];15(8):e0237959. Available from: https://journals.plos.org/plosone/article?id=10.1371/journal.pone.0237959

96. Apenteng OO, Osei PP, Oduro B, Kwabla MP, Ismail NA. The impact of implementing HIV prevention policies therapy and control strategy among HIV and AIDS incidence cases in Malaysia. Infectious Disease Modelling [Internet]. 2020 Jan 1 [cited 2024 May 28];5:755. Available from: /pmc/articles/PMC7536735/

97. Akdim K, Ez-Zetouni A, Zahid M. The influence of awareness campaigns on the spread of an infectious disease: a qualitative analysis of a fractional epidemic model. Modeling Earth Systems and Environment [Internet]. 2022 Mar 1 [cited 2024 May 28];8(1):1311–9. Available from: https://link.springer.com/article/10.1007/s40808-021-01158-9

98. Ahmad Z, Arif M, Ali F, Khan I, Nisar KS. A report on COVID-19 epidemic in Pakistan using SEIR fractional model. Scientific Reports 2020 10:1 [Internet]. 2020 Dec 17 [cited 2024 May 28];10(1):1–14. Available from: https://www.nature.com/articles/s41598-020-79405-9

99. Acuña-Zegarra MA, Santana-Cibrian M, Velasco-Hernandez JX. Modeling behavioral change and COVID-19 containment in Mexico: A trade-off between lockdown and compliance. Mathematical Biosciences. 2020 Jul 1;325:108370.

100. Abdin AF, Fang YP, Caunhye A, Alem D, Barros A, Zio E. An optimization model for planning testing and control strategies to limit the spread of a pandemic – The case of COVID-19. European Journal of Operational Research. 2023 Jan 1;304(1):308–24.

1.3. Additional Results

1.3.1. Modelling Field

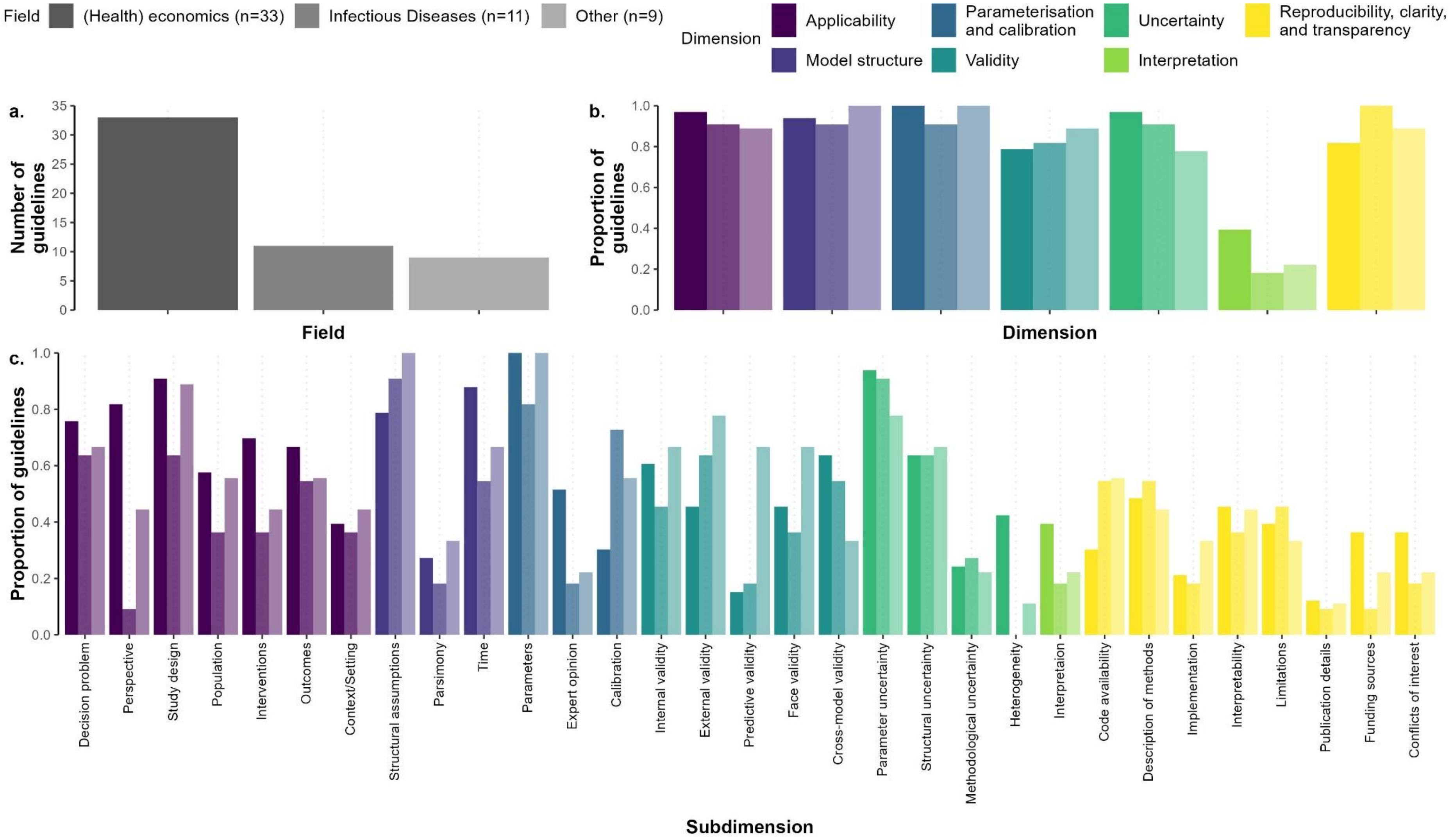

Figure S1.3.1. Prevalence of dimensions and subdimensions by field. (a) Number of guidelines stratified by field. (b) Overall proportion of guidelines (stratified by field of guideline) mentioning each dimension and (c) subdimension. The data used for these plots can be found in the Git repository linked in the data availability statement.

1.3.2. Overview of dimensions and subdimensions

1.3.2.1. Applicability

This dimension includes checklist items that are concerned with the aim or decision problem for a modelling study, and the applicability of individual elements of the modelling study to said aim or decision problem.

1.3.2.1.1. Decision problem

Guidelines covering this subdimension mentioned that the decision problem that a modelling study aims to answer should be clearly stated. Some guidelines went a step further to say that the relevance of the decision problem should be addressed, for example by relating it to a specific policy or pending decision.

1.3.2.1.2. Perspective

This subdimension included mentioning that the perspective (e.g. societal or a specific health economics/healthcare system) chosen by a study should be clearly stated.

1.3.2.1.3. Study design

This subdimension considers whether the design of a modelling study is described clearly and whether the appropriateness of the chosen design to achieve the study's aims is justified. This includes the type of model chosen, the scope of the model, and the analytical framework of the study, among other things.

1.3.2.1.4. Population

Guidelines covering this subdimension mentioned that the population considered in the modelling study (including all relevant demographic characteristics) should be clearly described and should be relevant to the decision problem of the study.

1.3.2.1.5. Interventions

This subdimension stated that modelling studies should consider relevant interventions and justify any exclusions. Health economic recommendations for interventions included elements such as either

considering *all* relevant competing treatment or prevention interventions which are currently used or may be used in the near future or considering the *main* alternative to the program in question.

1.3.2.1.6. Outcomes

Recommendations for this subdimension asked modellers to properly describe the outcomes considered or instructed readers to assess whether the outcomes considered were relevant to the stated decision problem and whether any relevant outcomes had been omitted.

1.3.2.1.7. Context/Setting

Guidelines covering this subdimension mentioned that the setting (geographical, environmental, etc.) and context (economic, political, infrastructural, etc.) of the decision problem should be well-described and well-represented in a modelling study. Several health economic guidelines emphasised the importance of defining the country in which a model is set, since healthcare options and costs can be very country dependent.

1.3.2.2. Model structure

This dimension is concerned with the description and justification of the model structure, including any underlying assumptions, chosen by the researchers conducting a modelling study.

1.3.2.2.1. Structural assumptions

This subdimension looked at the structural assumptions behind a model in a modelling study. Reporting guidelines mentioned that all structural assumptions should be explicitly and clearly reported, and quality assessment tools instructed the reader to assess whether structural assumptions were justified and consistent with existing knowledge about the phenomenon being modelled.

1.3.2.2.2. Parsimony

Having a model be as simple as possible but complex enough to incorporate all relevant interactions, with any simplifications being justified, was covered in this subdimension.

1.3.2.2.3. Time

This subdimension explicitly considered how different timeframes were considered and reported in a modelling study, and whether the choices were justified; this included the time horizon or study period the model was run for, as well as the cycle length or time step considered in the model.

1.3.2.3. Parameterisation and calibration

This dimension comprises considerations of the parameterisation of the model(s) in a modelling study, both directly from existing literature or data, or indirectly via calibration/model fitting.

1.3.2.3.1. Parameters

This subdimension included discussions of parameter values obtained from existing data, either directly from the literature or after transformation of data. This included the reporting of parameter values (including their sources or a justification), data sources, and data handling and transformation, and stating whether the parameter values obtained this way were credible.

1.3.2.3.2. Expert opinion

Discussing the use of expert opinion during model parameterisation was the focus of this subdimension. Recommendations on expert opinion often focused on the reporting of the methodology used to elicit such opinions. Other specific recommendations in this subdimension included stating that expert opinion, despite being a valid data source which may be crucial in the absence of alternative data sources, obtains the lowest data quality rating.

1.3.2.3.3. Calibration

This subdimension addressed the calibration of a model for the purposes of parameterisation. Recommendations focused on the sources of data used for calibration, the methodology chosen, and the presentation and discussion of the outcomes.

1.3.2.4. Validity

This dimension comprises suggestions about the reporting or quality of model validity and the validation process employed in a study.

1.3.2.4.1. Internal validity

Discussion about evidence and reporting of internal validity of the model(s) used in a study, i.e. the soundness of the mathematical logic of the model, the absence of bugs in the code used to implement the model, etc., was classified into this subdimension. Specific suggestions also included involving independent experts and sometimes stakeholders in a well-documented and reported review process of the model—including structure, parameterisation, and coding—to ensure that no errors are present. Replicating the model with different software was another suggested step for testing internal validity. Some reporting guidelines stated that more extensive details and outcomes of internal validation exercises should be reported in the appendices.

1.3.2.4.2. External validity

This subdimension included guidance about external validation, i.e. the validation of a model against independent data sets not used for model calibration. Most guidelines did not provide detailed guidance on how external validation should be addressed. However, some guidelines suggested including an explicit discussion of the theoretical relevance and generalisability of model results for various potential populations of interest beyond those included in the model. One guideline suggested that suitable data sources for external validation should be identified systematically, and that validation measures and results should be described in detail. Suggestions were sometimes contradictory across guidelines. For example, some guidelines mentioned that some data can be withheld from calibration to instead be used for validation while others recommended that all data should be used for parameterisation and calibration rather than being held back for validation.

1.3.2.4.3. Predictive validity

Predictive validity, arguably a type of external validity, is the ability of a model to accurately predict future occurrences. Guidelines covering this subdimension made suggestions about the evaluation of predictive validity, while simultaneously conceding that this is not always possible.

1.3.2.4.4. Face validity

This subdimension looked at whether the study and its results made intuitive sense. Reporting guidelines tended to emphasise the explanation of any counterintuitive results and the presentation

of outcomes of expert assessments of face validity and plausibility while quality assessment tools often instructed the reader to assess the face validity of the study for themselves.

1.3.2.4.5. Cross-model validity

This subdimension covered the validation of a model against other models or modelling studies, covering the identification and justification of the models used for comparison, the methodology used, and a discussion of the results, especially regarding any discrepancies or differences between the model results.

1.3.2.5. Uncertainty

This dimension comprised guidance about the handling of uncertainty in modelling studies, including assessments of the sensitivity of the results to this uncertainty. The subdimensions correspond to the different types of uncertainty present in such a study.

1.3.2.5.1. Parameter uncertainty

Parameter uncertainty, i.e. uncertainty due to imprecise parameter estimates, was the most mentioned subdimension of uncertainty. Guidelines that included this subdimension touched on the reporting of parameter uncertainty, sensitivity analysis methods to assess parameter uncertainty, and adequate discussion of the sensitivity analyses and uncertainty around results due to parameter uncertainty.

1.3.2.5.2. Structural uncertainty

This subdimension considered the assessment of uncertainty caused by structural assumptions, for instance by examining alternative model structures and conducting sensitivity analyses.

1.3.2.5.3. Methodological uncertainty

Guidelines covering this subdimension mentioned the assessment of uncertainty caused by methodological choices and assumptions made by the researchers in a modelling study.

1.3.2.5.4. Heterogeneity

This subdimension assessed uncertainty caused by heterogeneity in the study population and variability of results across different subgroups (e.g. different age groups or geographical regions) in the target population.

1.3.2.6. Interpretation

This dimension, consisting of a singular subdimension, is concerned with statements about whether the authors' interpretations of the results of a modelling study are unbiased, reasonable, substantiated by the results, and presented with a discussion of the limitations and caveats.

1.3.2.7. Reproducibility, clarity, and transparency

This dimension contains points of discussion and guidance pertaining to reproducibility of a modelling study, interpretability of the results by the target audience and laypeople, and transparency with respect to methods, funding, conflicts, and the limitations of the study.

1.3.2.7.1. Code availability

This subdimension touched upon public availability of code and technical documentation, mentioning that the model should be publicly available or made available upon request. Some guidelines stated that if the code cannot be made available, a reason for this should be given.

1.3.2.7.2. Description of methods

This subdimension focused on ensuring that the methods of a modelling study are described in enough detail to be reproducible. Some of the specific recommendations for this subdimension touched on describing any methods used to transform data, ensuring the study protocol is freely accessible, and providing the exact model equations.

1.3.2.7.3. Implementation

Clearly stating the software and programming languages used during a modelling study was the focus of this subdimension. Some guidelines went a step further, stating that the choice of software should be justified and that, where possible, widely available software should be used.

1.3.2.7.4. Interpretability

Guidelines covering this subdimension mentioned that the reporting of methods and results in a modelling study should be done in such a way as to be accessible to the target audience and any interested reader (especially policy makers) regardless of their technical background. This includes using non-technical language and documentation. At a minimum, a non-technical summary and conclusion should be available.

1.3.2.7.5. Limitations

This subdimension touched on the need for transparent discussion of the limitations (and strengths) of the modelling approach and results of a study.

1.3.2.7.6. Publication details

Suggestions for auxiliary details that should be included in the publication of a modelling study, such as the disclosure of author contributions and an identification of the type of study in the title or abstract, were classified into this subdimension.

1.3.2.7.7. Funding sources

Guidelines covering this subdimension stated that the funders of a modelling study and their role in the study should be clearly described.

1.3.2.7.8. Conflicts of interest

The clear disclosure of any conflicts of interest and steps taken to mitigate any such conflicts was the focus of this subdimension.

1.3.3. Prevalence of dimensions and subdimensions stratified by guideline

Table S1.3.3.1. Prevalence of dimensions stratified by reporting guidelines

Study	Reference Number	Applicability	Model structure	Parameterisation and calibration	Validity	Uncertainty	Interpretation	Reproducibility, clarity, and transparency
Pokutnaya 2023	62		X	X				X
Kunst 2023	56	X	X	X	X	X	X	
Kim 2023	26	X	X	X	X	X	X	X
Breeze 2023	51	X	X	X	X	X		X
Husereau 2022	38	X	X	X		X		X
*Zawadzki 2021	70	X	X	X	X	X		X
Pollett 2021	36	X	X	X	X	X		X
Slayton 2020	67	X	X	X	X	X		X
Grimm 2020	29	X	X	X	X	X		X
*den Boon 2019	37	X				X		X
*WHO 2018	30	X	X	X	X	X		X
Monks 2018	25	X	X	X				X
CADTH 2017	28	X	X	X	X	X	X	X
*Ultsch 2016	69	X	X	X	X	X		
Sanders 2016	64	X	X	X	X	X		X
STIKO 2016	24	X	X	X	X	X		X
Heintz 2016	73	X	X	X		X		X
Dahabreh 2016	46	X	X	X	X	X	X	X
*Marshall 2015	58	X	X	X	X	X		X
Haji Ali Afzali 2013	55			X	X			
Abuelezam 2013	41	X	X	X	X	X	X	X
Langer 2012	71	X	X	X		X	X	X
Caro 2012	40	X	X	X	X	X		X
Bennett 2012	3	X	X	X	X	X		X
Petrou 2011	61	X	X	X	X	X		

Study	Reference Number	Applicability	Model structure	Parameterisation and calibration	Validity	Uncertainty	Interpretation	Reproducibility, clarity, and transparency
Garnett 2011	27		X	X	X	X		X
*Lopez-Bastida 2010	57	X	X	X	X	X	X	X
von der Schulenburg 2008	54	X	X	X	X	X		X
Chaikledkaew 2008	48	X	X	X	X	X	X	X
Walter 2006	72	X	X	X		X		X
Philips 2006	31	X	X	X	X	X		
Drummond 2005	34	X		X		X		X
*Goeree 2004	39	X	X	X	X	X	X	X
Weinstein 2003	32	X	X	X	X	X		X
*Akehurst 2000	50	X	X	X	X	X		X
Nuijten 1998	33	X	X	X	X	X		X
Siegel 1996	65	X	X	X	X	X	X	X

Table showing the presence or absence of each dimension by reporting guideline. X's mark the articles which cover the respective subdimensions.
Reference numbers refer to the numeration used in the reference list of the main manuscript.
*Eight guidelines marked with an asterisk are part of both reporting guidelines and quality assessment tools since both aspects are covered in the guidelines.

Table S1.3.3.2. Presence of dimensions stratified by quality assessment tools

Study	Reference Number	Applicability	Model structure	Parameterisation and calibration	Validity	Uncertainty	Interpretation	Reproducibility, clarity, and transparency
Thompson 2022	68	X	X	X	X	X	X	X
*Zawadzki 2021	70	X	X	X	X	X		X
Burns 2021	19	X	X	X	X			X
*den Boon 2019	37	X				X		X
*WHO 2018	30	X	X	X	X	X		X
Mauskopf 2018	59	X	X	X	X	X		X
Rosello 2017	63	X	X	X	X	X		X
*Ultsch 2016	69	X	X	X	X	X		
Catala-Lopez 2016	47	X	X	X	X	X		X
Adarkwah 2016	45	X	X	X	X	X		X
Ramos 2015	42	X	X	X	X	X		X
*Marshall 2015	58	X	X	X	X	X		X
Silva 2014	66	X	X	X	X	X	X	X
Caro 2014	18	X	X	X	X	X	X	X
*Lopez-Bastida 2010	57	X	X	X	X	X	X	X
Kopec 2010	35	X	X	X	X	X		X
Abellan Perpinan 2009	49	X	X	X	X	X		
Evers 2005	53	X	X	X		X	X	X
*Goeree 2004	39	X	X	X	X	X	X	X
Soto 2002	44	X	X	X	X	X	X	X
Sculpher 2000	43	X	X	X	X	X	X	
McCabe 2000	60	X	X	X	X	X		X
*Akehurst 2000	50	X	X	X	X	X		X
Drummond 1996	52	X	X	X		X	X	X

Table showing the presence or absence of each dimension by quality assessment tools. X's mark the articles which cover the respective subdimensions. Reference numbers refer to the numeration used in the reference list of the main manuscript.

*Eight guidelines marked with an asterisk are part of both reporting guidelines and quality assessment tools since both aspects are covered in the guidelines.

Table S1.3.3.3. Presence of subdimensions stratified by reporting guidelines

Study	Reference Number	Applicability							Model Structure			Parameterisation and calibration			Validity					Uncertainty				Inter-pret-ation	Reproducibility, clarity, and transparency							
		Decision problem	Perspective	Study design	Population	Interventions	Outcomes	Context/Setting	Structural assumptions	Parsimony	Time	Parameters	Expert opinion	Calibration	Internal validity	External validity	Predictive validity	Face validity	Cross-model validity	Parameter uncertainty	Structural uncertainty	Methodological uncertainty	Heterogeneity	Interpretaion	Code availability	Description of methods	Implementation	Interpretability	Limitations	Publication details	Funding sources	Conflicts of interest
Pokutnaya 2023	62								X			X		X											X	X	X					
Kunst 2023	56	X	X	X	X	X		X	X		X	X	X	X	X	X	X	X	X	X	X			X								
Kim 2023	26	X	X	X	X	X	X	X	X		X	X						X		X	X			X		X	X		X			
Breeze 2023	51	X		X					X	X		X		X	X	X		X	X	X	X				X	X						
Husereau 2022	38	X	X	X	X	X	X	X			X	X								X	X		X		X	X		X	X	X	X	X
*Zawadzki 2021	70	X			X		X		X			X		X	X	X			X	X	X				X							
Pollett 2021	36	X		X	X		X		X		X	X				X	X		X	X	X	X			X	X		X	X	X		
Slayton 2020	67	X		X				X	X		X	X		X	X	X				X					X	X						
Grimm 2020	29	X		X	X		X	X	X		X	X		X		X				X						X						
*den Boon 2019	37	X																		X	X							X	X			
*WHO 2018	30	X	X	X		X	X	X	X	X	X	X	X	X		X		X	X	X	X				X	X		X	X			X
Monks 2018	25	X		X			X		X		X	X													X	X	X					
CADTH 2017	28	X	X	X	X	X	X	X	X	X	X	X	X	X	X	X		X	X	X	X	X	X	X	X	X		X	X		X	X
*Ultsch 2016	69		X	X			X		X	X	X	X		X		X			X	X	X	X										
Sanders 2016	64	X	X	X	X	X			X		X	X		X	X	X		X		X	X					X		X		X	X	X

Study	Reference Number	Applicability							Model Structure			Parameterisation and calibration			Validity					Uncertainty				Inter-pret-ation	Reproducibility, clarity, and transparency							
		Decision problem	Perspective	Study design	Population	Interventions	Outcomes	Context/Setting	Structural assumptions	Parsimony	Time	Parameters	Expert opinion	Calibration	Internal validity	External validity	Predictive validity	Face validity	Cross-model validity	Parameter uncertainty	Structural uncertainty	Methodological uncertainty	Heterogeneity	Interpretaion	Code availability	Description of methods	Implementation	Interpretability	Limitations	Publication details	Funding sources	Conflicts of interest
STIKO 2016	24			X		X	X		X		X			X		X		X	X	X	X	X			X							
Heintz 2016	73		X	X			X		X		X	X								X	X				X	X						
Dahabreh 2016	46	X	X	X	X	X	X		X	X	X	X	X	X	X	X	X	X	X	X			X	X	X			X				X
*Marshall 2015	58	X	X	X	X			X	X		X	X		X	X	X	X	X		X	X				X	X	X	X				
Haji Ali Afzali 2013	55											X		X	X	X		X	X													
Abuelezam 2013	41	X			X	X		X	X		X	X		X	X					X	X	X		X					X		X	
Langer 2012	71	X	X	X	X	X	X	X			X	X	X							X	X	X		X		X			X		X	
Caro 2012	40	X	X	X	X	X	X		X	X	X	X				X	X	X	X	X	X	X			X			X	X	X	X	
Bennett 2012	3	X	X	X		X	X		X	X	X	X	X	X	X		X	X	X	X	X	X	X		X		X	X				X
Petrou 2011	61	X	X	X	X	X	X	X			X	X	X		X	X	X	X	X	X	X		X									
Garnett 2011	27								X	X		X		X	X	X	X	X		X	X							X	X			
*Lopez-Bastida 2010	57	X	X	X	X	X	X	X	X		X	X							X	X	X			X				X	X		X	X
von der Schulenburg 2008	54	X	X	X	X	X	X		X		X	X	X		X	X			X	X						X	X	X	X			X
Chaikledkaew 2008	48	X	X	X	X	X	X	X	X		X	X	X		X			X	X	X				X			X	X	X		X	X
Walter 2006	72	X	X	X	X	X	X				X	X	X							X						X					X	
Philips 2006	31	X	X	X		X	X		X		X	X	X	X	X			X	X	X	X	X	X									
Drummond 2005	34	X		X								X								X			X					X				

Study	Reference Number	Applicability							Model Structure			Parameterisation and calibration			Validity					Uncertainty				Inter-pret-ation	Reproducibility, clarity, and transparency							
		Decision problem	Perspective	Study design	Population	Interventions	Outcomes	Context/Setting	Structural assumptions	Parsimony	Time	Parameters	Expert opinion	Calibration	Internal validity	External validity	Predictive validity	Face validity	Cross-model validity	Parameter uncertainty	Structural uncertainty	Methodological uncertainty	Heterogeneity	Interpretaion	Code availability	Description of methods	Implementation	Interpretability	Limitations	Publication details	Funding sources	Conflicts of interest
*Goeree 2004	39			X		X	X		X	X	X	X	X	X	X			X		X	X		X	X		X						
Weinstein 2003	32		X	X			X		X	X	X	X	X	X	X				X	X	X		X		X				X			
*Akehurst 2000	50				X				X	X	X	X			X	X			X	X	X	X				X		X				
Nuijten 1998	33	X	X	X	X			X	X		X	X	X					X	X	X					X		X					X
Siegel 1996	65	X	X	X	X	X	X	X	X		X	X	X		X				X	X	X		X	X		X	X	X	X			

Table showing the presence or absence of each subdimension by reporting guideline. X's mark the articles which cover the respective subdimensions. Reference numbers refer to the numeration used in the reference list of the main manuscript.
*Eight guidelines marked with an asterisk are part of both reporting guidelines and quality assessment tools since both aspects are covered in the guidelines.

Table S1.3.3.4. Presence of subdimensions stratified by reporting guidelines

Study	Reference Number	Applicability							Model Structure			Parameterisation and calibration			Validity					Uncertainty				Inter-pret-ation	Reproducibility, clarity, and transparency							
		Decision problem	Perspective	Study design	Population	Interventions	Outcomes	Context/Setting	Structural assumptions	Parsimony	Time	Parameters	Expert opinion	Calibration	Internal validity	External validity	Predictive validity	Face validity	Cross-model validity	Parameter uncertainty	Structural uncertainty	Methodological uncertainty	Heterogeneity	Interpretaion	Code availability	Description of methods	Implementation	Interpretability	Limitations	Publication details	Funding sources	Conflicts of interest
Thompson 2022	68			X	X		X		X	X	X	X			X	X		X	X	X				X		X			X			X
*Zawadzki 2021	70	X			X		X		X			X		X	X	X			X	X	X				X							
Burns 2021	19			X					X			X			X	X									X							
*den Boon 2019	37	X																		X	X							X	X			
*WHO 2018	30	X	X	X		X	X	X	X	X	X	X	X	X		X		X	X	X	X				X	X		X	X			X
Mauskopf 2018	59	X	X	X		X	X	X	X	X	X	X	X	X	X	X		X	X	X	X		X		X	X	X	X				
Rosello 2017	63	X		X		X	X	X	X			X		X		X				X	X					X		X				
*Ultsch 2016	69		X	X			X		X	X	X	X		X		X			X	X	X	X										
Catala-Lopez 2016	47	X	X	X	X	X			X		X	X							X	X						X			X	X	X	X
Adarkwah 2016	45		X	X		X			X		X	X			X					X	X	X	X			X				X	X	
Ramos 2015	42	X	X	X	X	X	X		X		X	X	X		X	X	X	X	X	X	X		X		X			X	X		X	
*Marshall 2015	58	X	X	X	X			X	X		X	X		X	X	X	X	X		X	X				X	X	X	X				
Silva 2014	66	X	X	X	X	X	X		X		X	X			X	X				X	X	X	X	X				X			X	X
Caro 2014	18			X	X	X	X	X	X			X			X	X	X	X		X	X			X		X			X		X	X
*Lopez-Bastida 2010	57	X	X	X	X	X	X	X	X		X	X							X	X	X			X				X	X		X	X
Kopec 2010	35			X					X			X	X	X	X		X	X	X	X							X					

Study	Reference Number	Applicability							Model Structure			Parameterisation and calibration			Validity					Uncertainty				Inter-pret-ation	Reproducibility, clarity, and transparency							
		Decision problem	Perspective	Study design	Population	Interventions	Outcomes	Context/Setting	Structural assumptions	Parsimony	Time	Parameters	Expert opinion	Calibration	Internal validity	External validity	Predictive validity	Face validity	Cross-model validity	Parameter uncertainty	Structural uncertainty	Methodological uncertainty	Heterogeneity	Interpretaion	Code availability	Description of methods	Implementation	Interpretability	Limitations	Publication details	Funding sources	Conflicts of interest
Abellan Perpinan 2009	49		X		X	X			X		X	X							X	X			X									
Evers 2005	53	X	X	X	X	X	X				X	X								X				X								X
*Goeree 2004	39			X		X	X		X	X	X	X	X	X	X			X		X	X		X	X		X						
Soto 2002	44	X	X	X		X	X	X	X		X	X	X		X	X	X	X	X	X				X	X	X	X	X	X		X	X
Sculpher 2000	43	X	X	X				X	X		X	X	X		X	X			X	X				X								
McCabe 2000	60	X		X				X	X	X		X	X		X	X	X	X	X		X							X				
*Akehurst 2000	50				X				X	X	X	X			X	X			X	X	X	X				X		X				
Drummond 1996	52	X	X	X		X	X		X		X	X								X	X	X		X					X			

Table showing the presence or absence of each subdimension by quality assessment tools. X’s mark the articles which cover the respective subdimensions. Reference numbers refer to the numeration used in the reference list of the main manuscript.

*Eight guidelines marked with an asterisk are part of both reporting guidelines and quality assessment tools since both aspects are covered in the guidelines.
